# Acacetin protects against depression-associated dry eye disease by regulating ubiquitination of NLRP3 through gp78 signal

**DOI:** 10.3389/fphar.2022.984475

**Published:** 2022-10-10

**Authors:** Mingxia Xie, Hanqing Wang, Jun Peng, Dongqin Qing, Xi Zhang, Dongwei Guo, Pan Meng, Zhihong Luo, Xiaoye Wang, Qinghua Peng

**Affiliations:** ^1^ College of Clinical Medicine, Hunan University of Chinese Medicine, Changsha, Hunan, China; ^2^ College of Traditional Chinese Medicine, Hunan University of Chinese Medicine, Changsha, Hunan, China; ^3^ College of Pharmacy, Hunan University of Chinese Medicine, Changsha, Hunan, China; ^4^ The First Hospital of Hunan University of Chinese Medicine, Changsha, Hunan, China

**Keywords:** dry eye disease, depression, NLRP3 ubiquitination, gp78, acacetin

## Abstract

Dry eye disease (DED) is a multifactorial syndrome that commonly occurs with depression. However, therapies targeting depression-related dry eye disease are rare. In the current study, we studied the beneficial effect of a natural flavone, acacetin, in depression-associated dry eye disease by utilizing the chronic unpredictable mild stress (CUMS) depression model. Our data showed that acacetin improved the depressive behaviors in sucrose preference test (SPT), tail suspension test (TST) and forced swim test (FST); relieved the dry eye symptoms including corneal epithelial impairments, tear production decrease and goblet cell loss in CUMS mice. Acacetin also inhibited NOD-like receptor protein 3 (NLRP3) inflammasome expression levels and suppressed inflammatory responses *via* enhancing glycoprotein 78 (gp78)/Insulin induced gene-1 (Insig-1)-controlled NLRP3 ubiquitination in CUMS mice. Furthermore, knockdown of gp78 compromised acacetin-conferred protective efficacy in depression-related dry eye disease. In summary, our findings indicated that acacetin exerts beneficial effect in depression-associated dry eye disease, which is tightly related to gp78-mediated NLRP3 ubiquitination.

## Introduction

Depression is one of the most prevalent and disabling mental disorder characterized by sadness, anhedonia, and a feeling of worthlessness (Hao et al., 2019). According to the World Health Organization (WHO), 350 million people worldwide suffer from depression, and it is estimated to be a major cause of disability by 2030 (Wang et al., 2019). Dry eye disease is defined as a “multifactorial disease of the tears and ocular surface that results in symptoms of discomfort, visual disturbance, and tear film instability with potential damage to the ocular surface (Messmer, 2015). Previous researches demonstrate that it frequently occurs with depression (Zhang et al., 2019; Zhou et al., 2022). However, the treatment for depression-associated dry eye disease has not been fully studied.

Inflammasomes are a group of cytosolic protein complexes that underlies a wide variety of disease (Schnappauf et al., 2019; Abbate et al., 2020). Existing evidence suggest that the NLRP3 inflammasome plays an important role in the pathological process and treatment of depression and dry eye disease (Arioz et al., 2019; Park et al., 2019). The NLRP3 inflammasome is an oligomeric complex comprised of the sensors NLRP3, the adaptor apoptosis-associated speck-like protein containing a CARD (ASC) and the effector protein-Caspase-1. In the presence of immune activators such as pathogen-associated molecular patterns (PAMPs), danger-associated molecular patterns (DAMPs), or other exogenous invaders, the pyrin domains of NLRP3 binds to that of ASC, and subsequently the caspase recruitment domain of ASC recruits and interacts with pro-caspase-1 (Shao et al., 2015). These interactions form the NLRP3 inflammasome and promote the autocatalytic cleavage of pro-caspase-1 (Luo et al., 2017). Thereafter, the activated caspase-1 converses pro–interleukin (IL)-1 β and pro-IL-18 to bioactive IL-1β and IL-18, respectively, and cleaves the gasdermin D (GSDMD) to generate the N-terminal fragment to induce pore formation, cytokine release and pyroptotic cell death (Sharif et al., 2019; Wang et al., 2021). Recently, the membrane-bound E3 ubiquitin ligase gp78 has been demonstrated to mediate the ubiquitination (a key determinator of protein fate by tagging proteins for proteasomal degradation) of NLRP3 *via* Insig-1, which ultimately affects the activation of NLRP3 inflammasome (Xu et al., 2022).

Acacetin (5,7-dihydroxy-4′-methoxyflavone) is a natural flavone present in a variety of plants, for instance, Turnera diffusa and Saussurea involucrate (Singh et al., 2020). A broad spectrum of pharmacological and biochemical activities, e.g., antioxidant, anti-inflammatory and neuroprotective effects, have been detected in acacetin (Xiao et al., 2019; Singh et al., 2020; Wu et al., 2021). Acacetin suppresses activation of the NLRP3 inflammasome in mice with cerebral ischemia-reperfusion injury (Bu et al., 2019). Moreover, chronic administration of acacetin exerts evident antidepressant-like efficacy in mice (Xiao et al., 2019). However, the effect of acacetin in depression-related dry eye disease remains unknown. Thus, in this study we investigated the function of acacetin in depression-associated dry eye disease and the underlying mechanisms with the usage of CUMS mice.

## Materials and methods

### Animals and drug treatment

Male C57BL/6 mice, weighing 18–22 g, were purchased from Changzhou Cavens Laboratory Animal Co., Ltd. All animal procedures were carried out in accordance with Hunan University of Chinese Medicine Medical Ethical committee (NO. 2021.22). Animals were housed on a 12-h light/dark cycle (lights on at 6 a.m.) at room temperature of 22 ± 1°C, with access to food and water *ad libitum*.

Following a 1-week acclimatization period for the mice, the animals were randomly assigned to five groups: Control (non-stress + normal saline treatment); Vehicle (CUMS+normal saline treatment); Acacetin-L (CUMS+5 mg/kg acacetin treatment); Acacetin-H (CUMS+15 mg/kg acacetin treatment); Escitalopram (CUMS +10 mg/kg escitalopram treatment). Acacetin was purchased from Sigma-Aldrich (00,017) and dissolved in normal saline. The CUMS groups were singly housed and exposed to two unpredictable stressors each day for 7 weeks according to a method previously described with minor modifications (Zhang et al., 2019; Xu J et al., 2020). The stressors included: 24 h food deprivation; 24 h water deprivation; 24 h wet bedding (100 g sawdust bedding added with 200 ml water); 24 h no bedding; 24 h cage tilting; 6 h 50 ml centrifuge tube confinement; 15 min cage shaking; 5 min tail pinching; overnight illumination (twice per week). The unstressed controls were group housed under normal conditions. At 4 weeks after the starting of CUMS modeling, the sucrose preference ratio and tear production were tested (Supplementary Figure S1). Acacetin (5, 15 mg/kg/day) or escitalopram (10 mg/kg/day) was intragastrically administrated once daily for 3 consecutive weeks starting at the 5th week of CUMS modeling. Control and vehicle groups received equal volume of normal saline administration. Afterwards, behavioral assessment including sucrose preference test (SPT), tail suspension test (TST), forced swim test (FST), and open field test (OFT) was conducted, and the development of dry eye disease was evaluated. Finally, mice were sacrificed for tissue collection at the end of the experiment. For the gp78 knockdown experiment, 100 ul of gp78 siRNAs (Universal Biosystems (Anhui) Co., Ltd.; Interfering sequence: 5′-AGC​TTA​TCC​AGT​GTA​TTG​TGT-3’; Sense strand siRNA: 5′-AGC​UUA​UCC​AGU​GUA​UUG​UGU​tt-3’; Antisense strand siRNA: 5′-ACA​CAA​UAC​ACU​GGA​UAA​GCU​tt-3′) was infected to mice through vein injection 4 weeks after the starting of CUMS modeling.

### Sucrose preference test

The SPT was performed to assess anhedonia as described previously (Li et al., 2021). Mice were first habituated to 1% sucrose for 3 days. After food and water deprivation for 24 h, each mouse was provided with two bottles, one containing 1% sucrose solution, and the other containing drinking water. The test lasted for 24 h, and the positions of the bottles were switched in the middle of the test to avoid side preference. Sucrose preference ratio was calculated by using the following formula: sucrose preference (%) = sucrose intake/(sucrose intake + water intake) × 100%.

### Tail suspension test and forced swim test

Behavioral despair of mice was evaluated by the TST and FST as reported previously (Abdelhamid et al., 2014; Li et al., 2021).

For TST, the mouse was hung on a suspension bar (30 cm above the apparatus floor) by attaching an adhesive tape to a position 1 cm from the tip of its tail. The test lasted for 6 min, and the immobility time of the last 4 min was analyzed by ANY-maze software.

For FST, each mouse was placed in a glass cylinder (20 cm height, 15 cm diameter) filled with water (23 ± 1°C). Mice were forced to swim for 6 min, and the amount of time spent immobile during the last 4 min was analyzed by ANY-maze software.

### Open field test

Locomotor activity was evaluated using the OFT. Each mouse was placed in the center of the open-field apparatus (40 × 40 × 25 cm) and allowed to freely explore the open area for 5 min. Distance traveled by each animal was analyzed using ANY-maze software. The apparatus was cleaned with 75% ethanol after each trial (Shen et al., 2021).

### Measurement of tear production

Tear production was examined using cotton phenol red threads (Jingming, Tianjin, China) as previous report (Fakih et al., 2019). In brief, the top of the thread (yellow) was placed on the lower palpebral conjunctiva for 20 s. After absorbing tears, the color of the thread would change to red. Tear production was determined by measuring the length of the wet portion of the thread.

### Fluorescein staining

To examine corneal epithelial defects, 1 µl of 1% sodium fluorescein was instilled onto the right eye of anaesthetized mice using a micro-pipette (Musayeva et al., 2021). Ocular surface staining was then observed using a slit lamp under a cobalt blue light. Quantification of the corneal defect area was carried out by using the following formula: Corneal defect area (%) = (fluorescein sodium positive area/the whole cornea) × 100%

### Periodic acid-schiff and nissl staining

The specimens were fixed in 4% paraformaldehyde, embedded in paraffin, and cut into 5-μm-thick sections. The sections were stained with periodic acid-Schiff (ab150680, Abcam) for goblet cells or Nissl staining solution (C0117, Beyotime) to identify Nissl bodies according to the manufacturer’s instructions. Subsequently, the slices were dehydrated in a gradient ethanol, cleared in xylene, and covered with neutral resins. The images were acquired by a microscope and analyzed with ImageJ.

### Immunohistochemistry

The immunohistochemical staining was carried out according to a previous protocol (Liu et al., 2021). Briefly, tissue sections were fixed in 4% paraformaldehyde for 20 min, and permeabilized with 0.5% Triton X-100 for 10 min. Nonspecific antibody binding was blocked by 3% BSA for 1 h. The samples were then incubated with anti-NLRP3 (ab270449, Abcam) overnight at 4°C, followed by the incubation of secondary antibody for 1 h at room temperature. Positive cells were detected by 3,3′-diaminobenzidine (DAB) staining, and the nuclei was counterstained with hematoxylin. Finally, the slices were cleared with xylene, mounted by neutral resin, and observed under a microscope.

### Western blot

Western blotting assays were performed as previously described (Xu X et al., 2020). For protein extraction, hippocampus or cornea samples were homogenized in ice-cold RIPA lysis buffer supplemented with protease and phosphatase inhibitors. Homogenates were centrifuged at 12,000×g for 15 min at 4°C, and supernatants were collected. The concentrations of proteins were quantified by bicinchoninic acid (BCA) protein assay, and proteins were denatured by boiling for 5 min. Equal amounts of protein samples were subjected to electrophoresis on a sodium dodecyl sulfate (SDS)-polyacrylamide gel electrophoresis (PAGE) gels, followed by being transferred onto polyvinylidene difluoride (PVDF) membranes. After being blocked with 3% bovine serum albumin (BSA) for 1 h, the membranes were probed with primary antibodies including anti-gp78 (ab227450, Abcam), anti-Insig-1 (55282-1-AP, Proteintech), anti-NLRP3 (ab270449, Abcam), anti-Caspase-1 (AF5418, Affinity), anti-Cleaved-Caspase-1 (AF4022, Affinity), anti-gasdermin D (GSDMD)-N (DF13758, Affinity), anti-GSDMD (AF4012, Affinity) and anti-GAPDH (10494-1-AP, Proteintech) overnight at 4°C, and then incubated with secondary antibodies for 1 h at room temperature. Afterwards, the protein bands were visualized with enhanced chemiluminescence (ECL) detection system. Protein expression levels were analyzed using ImageJ software and normalized to GAPDH.

### Co-immunoprecipitation

The immunoprecipitation was conducted following a previously reported protocol (Lin et al., 2017). *Hippocampus* tissues were homogenized in ice-cold NP-40 lysis buffer supplemented with protease and phosphatase inhibitors. Insoluble debris was removed by centrifugation at 12,000×g for 15 min at 4°C. Protein concentrations were determined using the BCA method. Tissue lysates containing 500 μg of total protein were incubated with anti-NLRP3 overnight at 4°C with constant rotation. Next, 50 μl of protein A/G agarose beads was added to incubate for 4 h. The beads were extensively washed and boiled in SDS loading buffer for 5 min. The ubiquitination of NLRP3 was evaluated in the western blot analysis by using Ubiquitin antibody (3936, Cell Signaling Technology).

### Enzyme-linked immunosorbent assay

Sample preparation: Tears were collected from the lateral canthus of mice using glass microcapillary tubes. One sample consisted of tears from both eyes of one mouse that were pooled in phosphate buffered saline (PBS) + 0.1% BSA and stored at −80°C until the assay was performed (Dogru et al., 2022). *Hippocampus* tissues of mice were isolated, snap-frozen in liquid nitrogen, and stored at − 80°C for protein extraction. The tissues rinsed with cold PBS to remove residual blood, weighed, and added with PBS containing protease inhibitor cocktail (1:9 w/v). Samples were then homogenized, centrifuged at 3500 ×g for 20 min, and supernatants were collected (Xue et al., 2021; Dang et al., 2022).

The levels of inflammatory cytokines including tumor necrosis factor (TNF)-α, IL-1β, and IL-18 in hippocampi and tears were detected using Mouse TNF-α ELISA Kit (PT512, Beyotime), Mouse IL-1β ELISA Kit (PI301, Beyotime), and Mouse IL-18 ELISA Kit (PI553, Beyotime), respectively, as per company instructions.

### Statistical analysis

Statistical analyses were completed in GraphPad Prism 7.0 software. All results are presented as the mean ± standard error of the mean (SEM) values. Differences between two groups were evaluated by Student’s *t*-test. One-way analysis of variance (ANOVA) was used when comparing multiple groups. *P* < 0.05 was considered statistically significant.

## Results

### Acacetin attenuates depression-like behavior in chronic unpredictable mild stress mice

**FIGURE 1:**
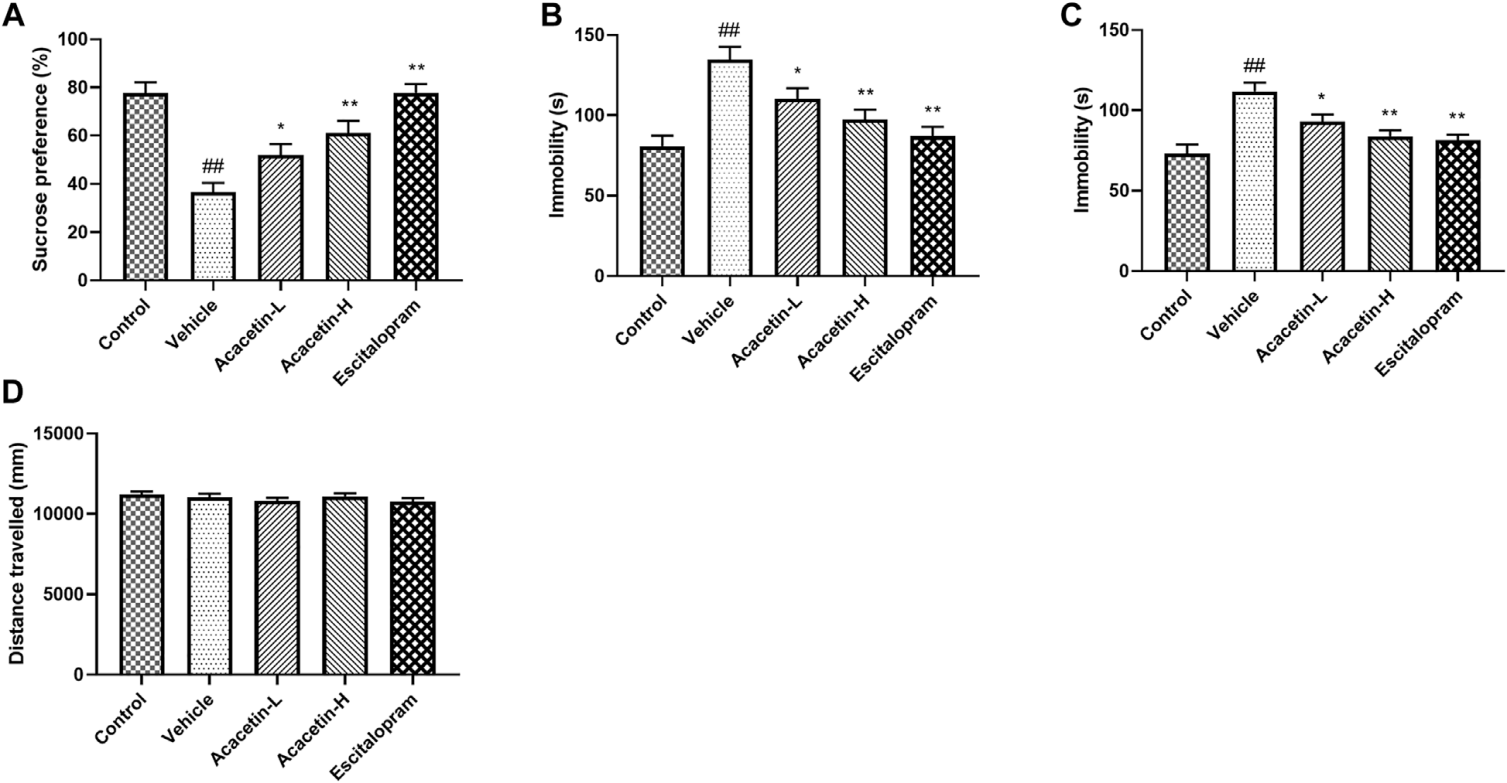
Acacetin attenuates depression-like behavior in chronic unpredictable mild stress (CUMS) mice. **(A)** The preference ratio of mice in the sucrose preference test. **(B)** Immobility duration of mice in the tail suspension test. **(C)** Immobility duration of mice in the forced swim test. **(D)** Total distance travelled in the open field test. Results are expressed as the mean ± standard error of the mean (SEM; *n* = 9 per group). ^##^ *p* < 0.01 vs. Control; *p < 0.05, **p < 0.01 vs. Vehicle.

Depressive behavior was assessed on all mice using SPT, TST and FST tests (Figures 1A–C). The stressed vehicles displayed higher sucrose preference ratio in SPT and longer immobility time in both TST and FST compared to unstressed controls, indicating the development of depression following CUMS. Acacetin or escitalopram, however, alleviated CUMS-induced depressive symptoms above. No differences were observed on the locomotor activity indicated by the total distance travelled in the OFT test (Figure 1D).

### Acacetin improves dry eye disease in chronic unpredictable mild stress mice

**FIGURE 2:**
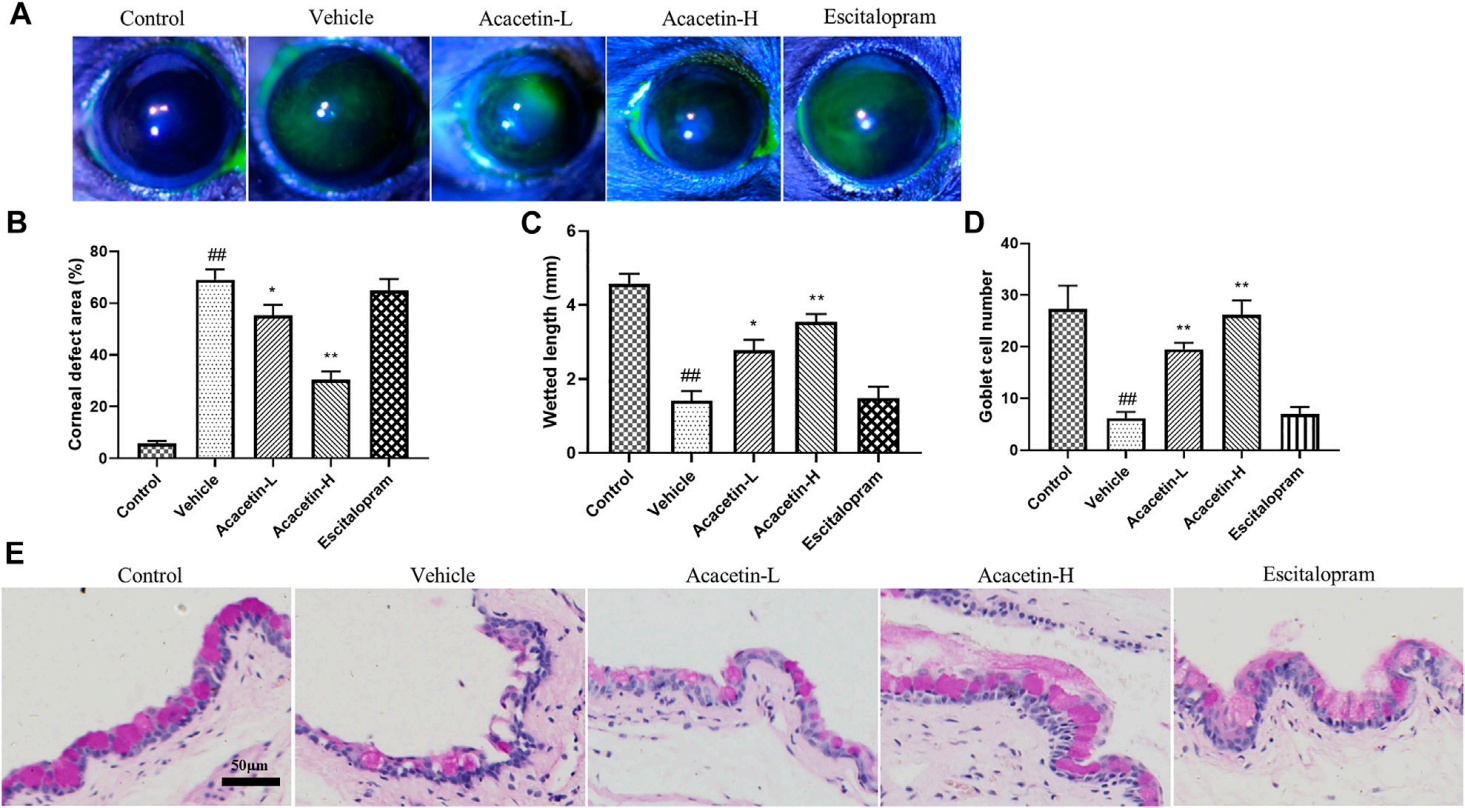
Acacetin improves dry eye symptoms in CUMS mice. **(A–B)** Determination of corneal epithelial defects by fluorescein staining (*n* = 9 per group). **(C)** Tear volumes of mice were measured using cotton phenol red threads (*n* = 9 per group). **(D–E)** The number of goblet cells was determined by periodic acid-Schiff (PAS) staining (*n* = 6 per group). Results are expressed as the mean ± SEM. ^##^ *p* < 0.01 vs. Control; *p < 0.05, **p < 0.01 vs. Vehicle.

Next, we assessed whether acacetin would be an effective therapy for CUMS-associated dry eye disease. Corneal fluorescein staining was used to examine corneal epitheliopathy after exposure to CUMS. As shown in Figures 2A–B, almost no stained spots were found in the cornea of control mice, whereas large number of stained spots were observed in the corneal surface of vehicle-treated mice, indicating corneal damage following CUMS. Tear production and goblet cells (responsible for the release of tear-stabilizing mucin) were assessed by cotton phenol red threads and periodic acid-Schiff (PAS) staining, respectively (Puro, 2020). As shown in Figures 2C–E, CUMS resulted in reduced wetted length and goblet cells in mice. Interestingly, acacetin, but not escitalopram, rescued CUMS-induced corneal defects, tear reduction, and loss of goblet cells in mice. These results suggest that acacetin protects depressive mice from dry eye disease.

### Acacetin deactivates NOD-like receptor protein 3 inflammasome *via* NOD-like receptor protein 3 ubiquitination in depression-related dry eye disease

**FIGURE 3:**
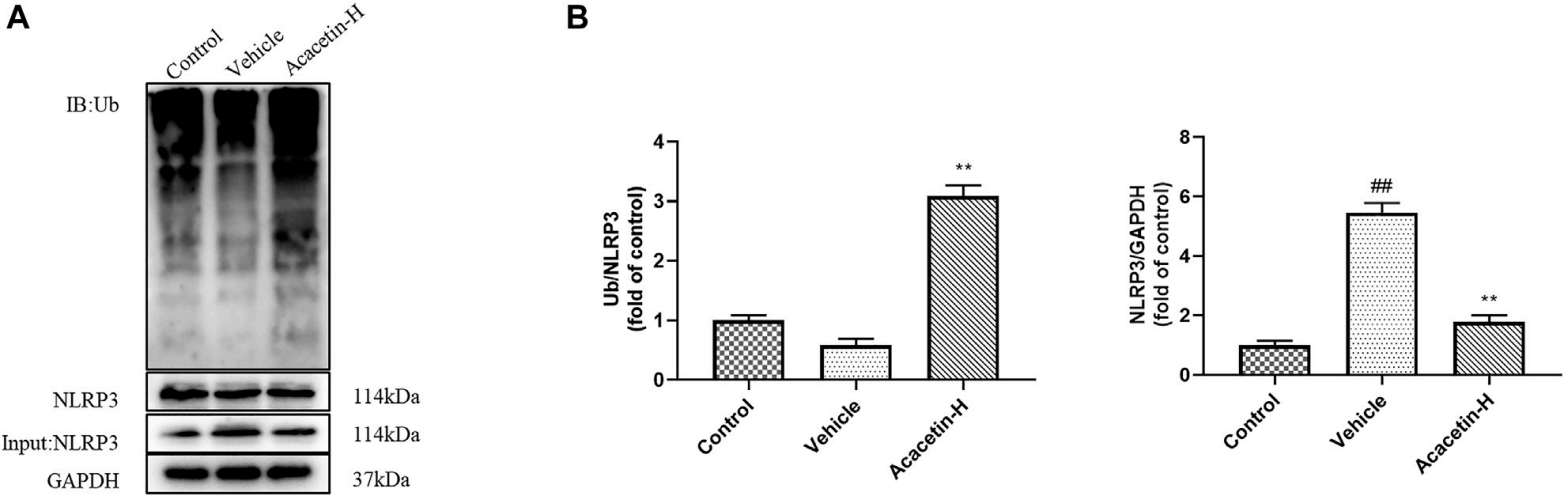
Effect of acacetin on NLRP3 ubiquitination in the hippocampus. **(A)** The ubiquitination of NLRP3 in the hippocampus of mice was detected by co-immunoprecipitation (co-ip). **(B)** Quantification of ubiquitinated NLRP3 expression levels and NLRP3 protein expression in the hippocampus of mice. Results are expressed as the mean ± SEM (*n* = 3 per group). ^##^ *p* < 0.01 vs. Control; **p < 0.01 vs. Vehicle.

**FIGURE 4:**
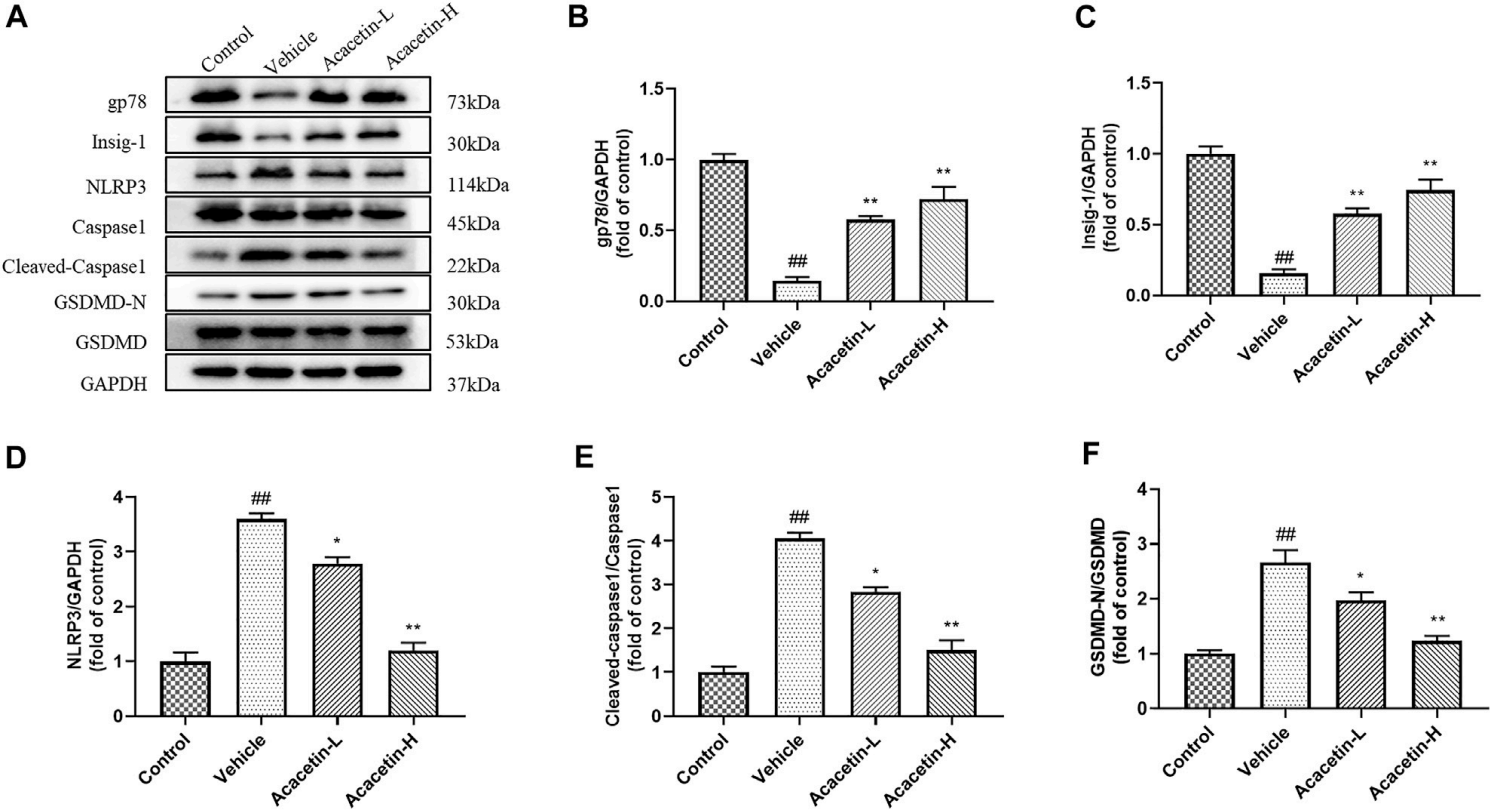
Effect of acacetin on gp78-mediated NLRP3 inflammasome in the hippocampus. **(A)** Representative western blots of gp78, Insig-1, NLRP3, Caspase1, cleaved Caspase1, GSDMD-N, and GSDMD. **(B–F)** Quantitative analysis of western blot results. Results are expressed as the mean ± SEM (*n* = 3 per group). ^##^ *p* < 0.01 vs. Control; *p < 0.05, **p < 0.01 vs. Vehicle.

**FIGURE 5:**
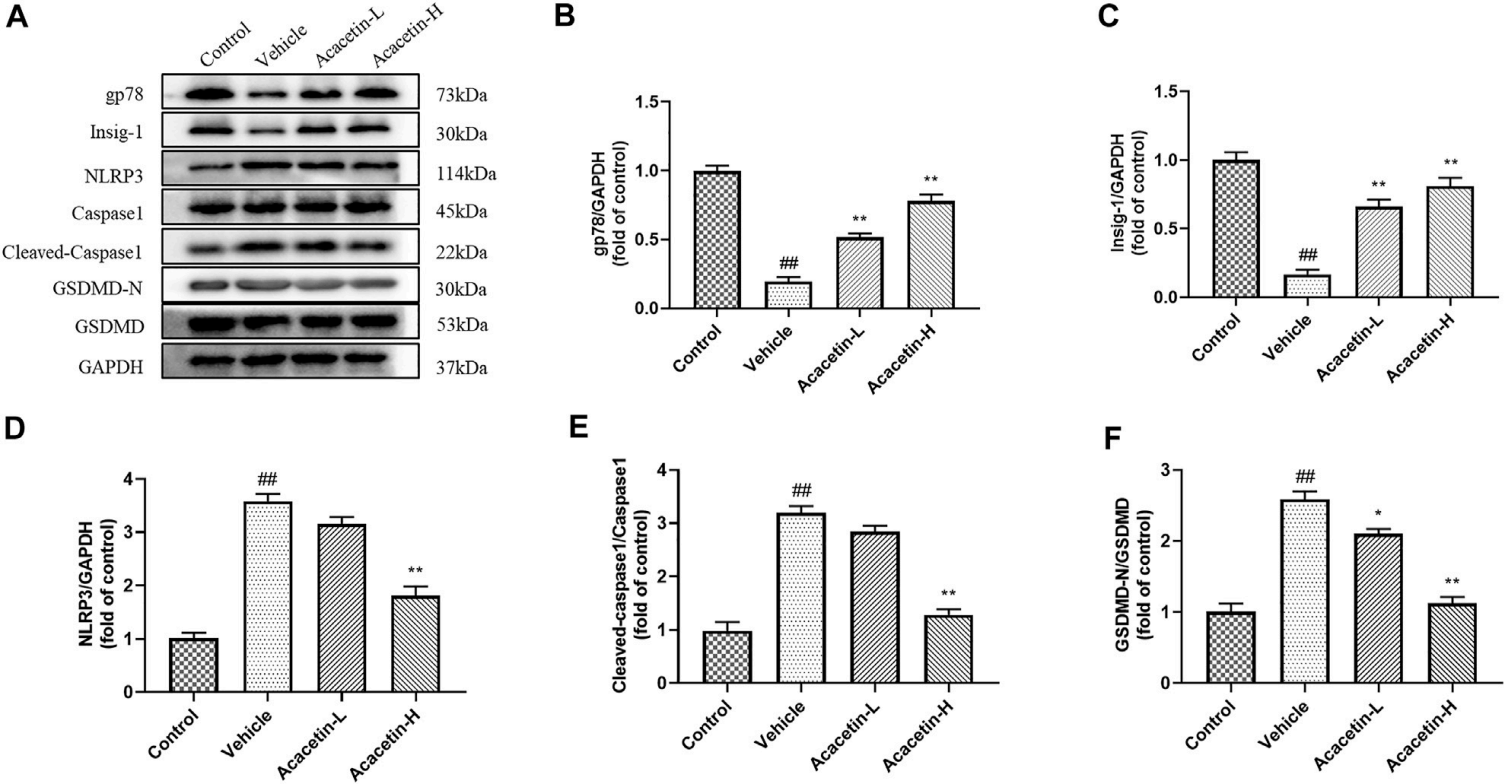
Effect of acacetin on gp78-mediated NLRP3 inflammasome in the cornea. **(A)** Representative western blots of gp78, Insig-1, NLRP3, Caspase1, cleaved Caspase1, GSDMD-N, and GSDMD. **(B–F)** Quantitative analysis of western blot results. Results are expressed as the mean ± SEM (*n* = 3 per group). ^##^ *p* < 0.01 vs. Control; *p < 0.05, **p < 0.01 vs. Vehicle.

**FIGURE 6:**
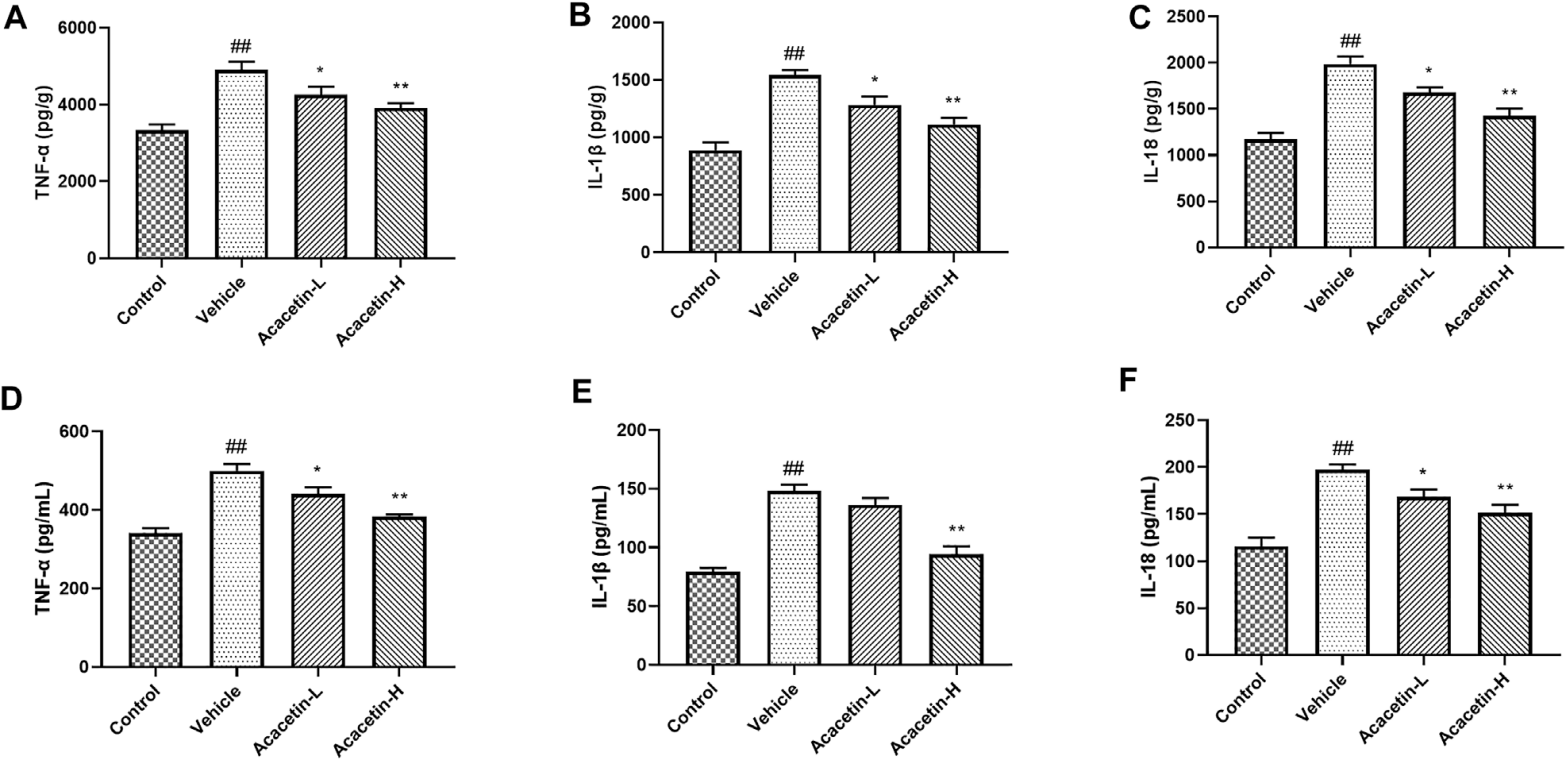
ELISA analyses of inflammatory factors in the hippocampus and tears. **(A–C)** Production of TNF-α, IL-1β, and IL-18 in the hippocampus of mice. **(D–F)** Concentrations of TNF-α, IL-1β, and IL-18 in the tears of mice. Results are expressed as the mean ± SEM (*n* = 6 per group). ^##^ *p* < 0.01 vs. Control; *p < 0.05, **p < 0.01 vs. Vehicle.

We then determined the involvement of NLRP3 ubiquitination-mediated NLRP3 inflammasome activation in the protective effect of acacetin. The co-ip results revealed that the CUMS modeling decreased ubiquitinated NLRP3 expression (though did not reach significance), and significantly increased NLRP3 protein levels in vehicle-treated mice (Figure 3). Interestingly, acacetin intervention greatly enhanced NLRP3 ubiquitination and suppressed NLRP3 protein levels in CUMS mice. Additionally, the western blotting and ELISA assays demonstrated that with the activation of gp78/Insig-1 signal, acacetin promoted hippocampal and corneal NLRP3 inflammasome (NLRP3, cleaved-Caspase1, GSDMD-N) protein expression, and boosted the production of proinflammatory factors (TNF-α, IL-1β, and IL-18) in hippocampi and cornea (Figures 4–6). Collectively, these findings demonstrated that acacetin deactivates NLRP3 inflammasome *via* gp78/Insig-1-mediateed NLRP3 ubiquitination in CUMS-induced dry eye disease.

### Knockout of glycoprotein 78 compromised acacetin-elicited protection in depression-related dry eye disease

**FIGURE 7:**
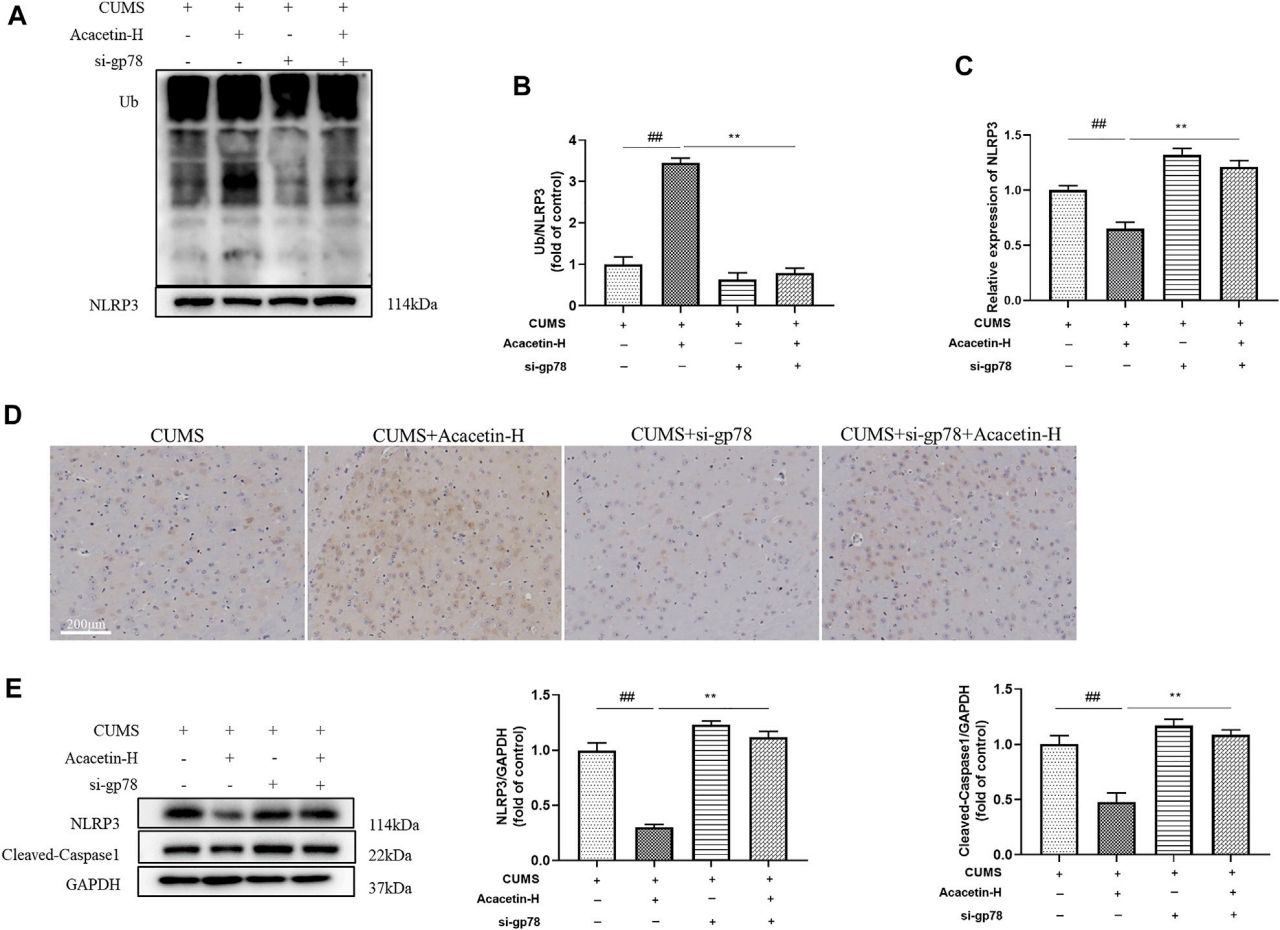
Influence of gp78 siRNAs on hippocampal NLRP3 inflammasome in CUMS mice. **(A–B)** NLRP3 ubiquitination levels in the hippocampus of mice were determined by co-ip. **(C–D)** Hippocampal NLRP3 expression levels of the experimental groups were detected with the immunohistochemistry. **(E)** Protein expression of NLRP3 and Cleaved-Caspase1 in the western blotting. Results are expressed as the mean ± SEM (*n* = 3 per group).

**FIGURE 8:**
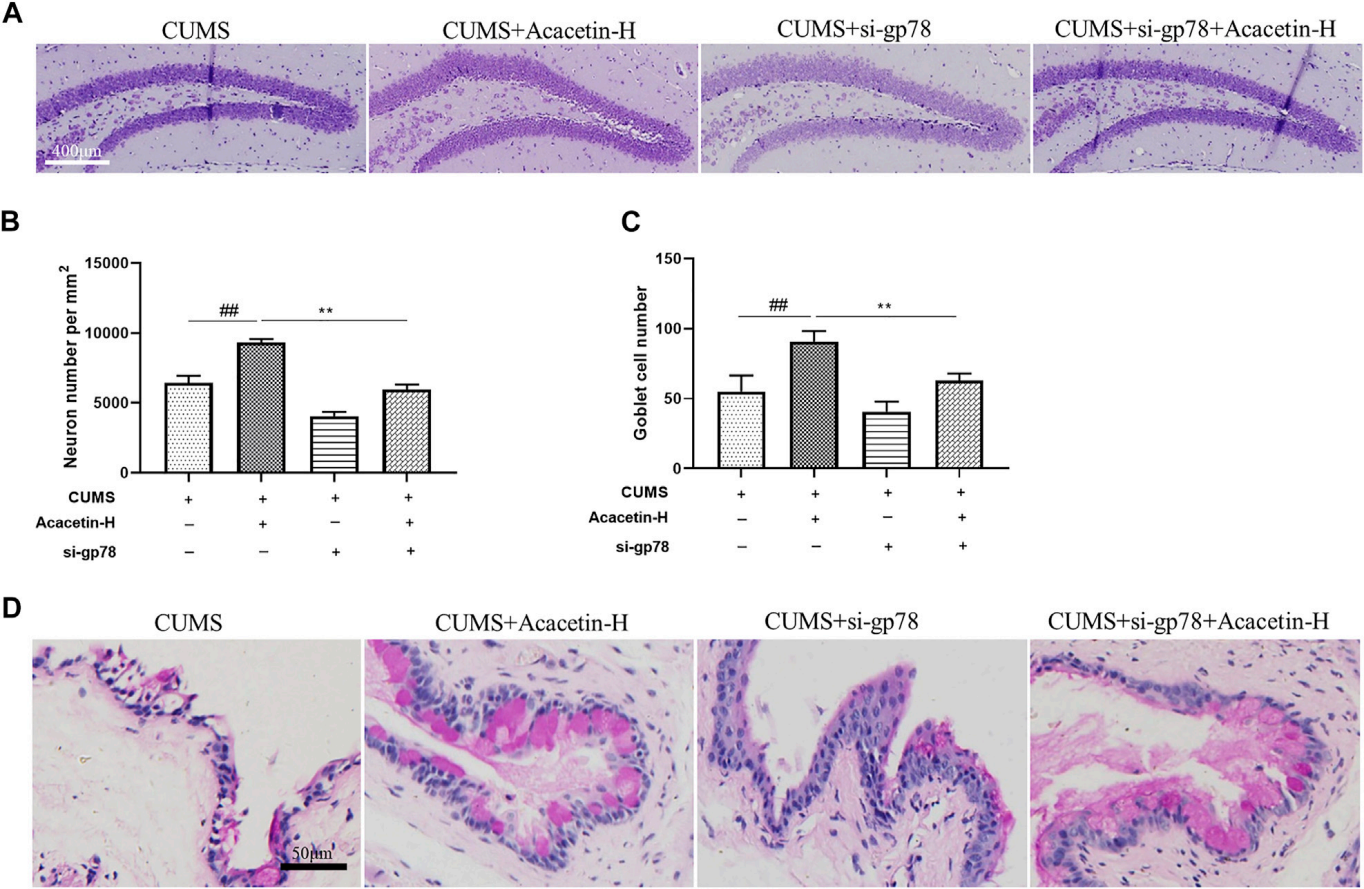
Influence of gp78 siRNAs on hippocampus Nissl bodies and goblet cells in CUMS mice. **(A–B)** Representative images of Nissl staining and quantitative analysis of Nissl bodies in the hippocampus. **(C–D)** Representative images of PAS staining and quantitative analysis of goblet cells in the cornea. Results are expressed as the mean ± SEM (*n *= 3 per group).

**FIGURE 9:**
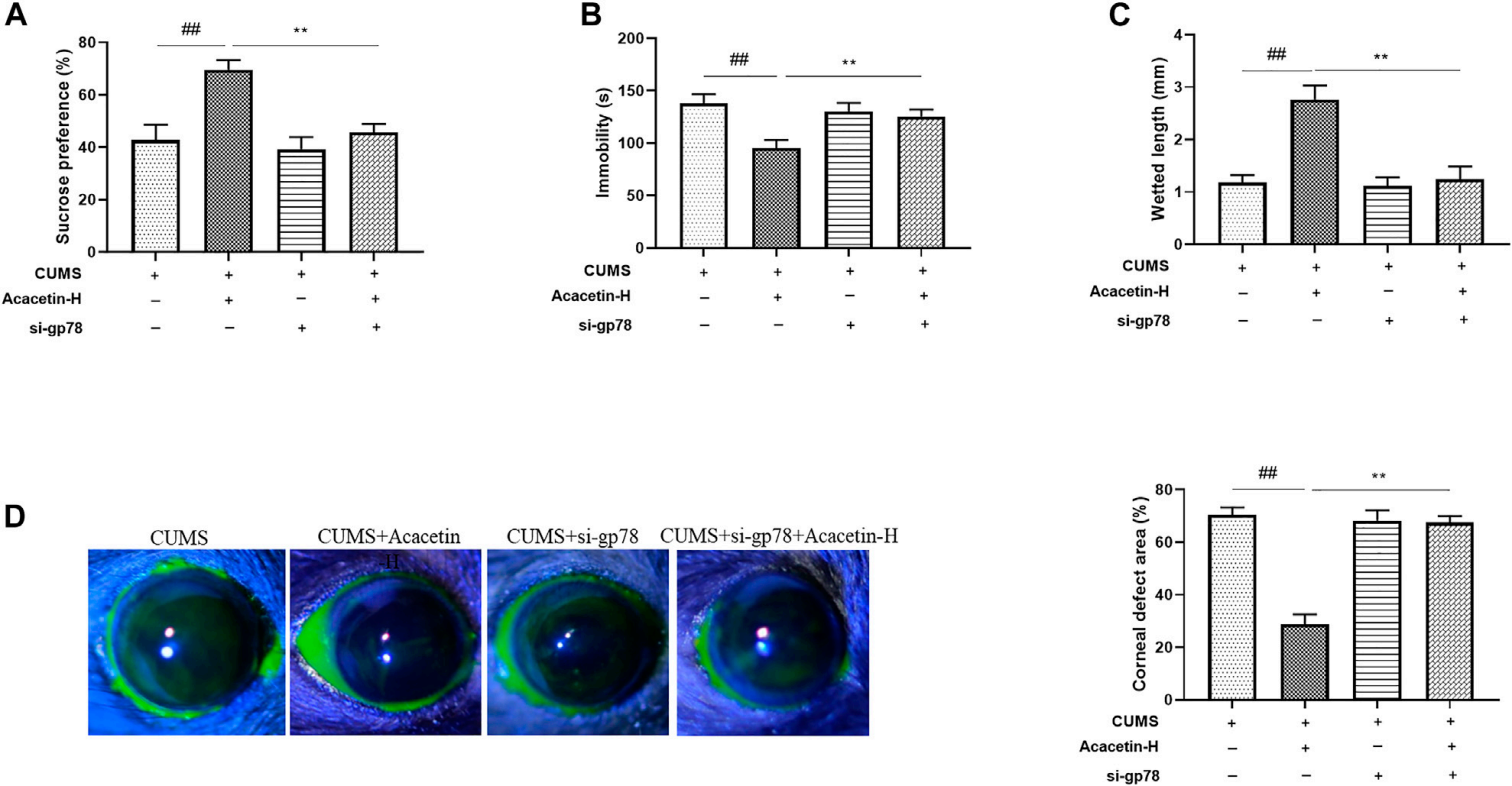
Impact of gp78 siRNAs on depressive behaviors and dry eye symptoms in CUMS mice. **(A)** Sucrose preference percentage of mice in the sucrose preference test. **(B)** Immobility time of mice in the tail suspension test. **(C)** Tear volumes of mice were measured using cotton phenol red threads. **(D)** Determination of corneal epithelial defects by fluorescein staining in CUMS mice. Results are expressed as the mean ± SEM (*n* = 9 per group).

To elucidate the role of gp78 in acacetin-conferred beneficial effect, we injected CUMS mice with gp78 siRNAs (Supplementary Figure S2). As shown in Figures 7,8, gp78 siRNA infection greatly inhibited the increase of NLRP3 ubiquitination in CUMS mice resulted from acacetin treatment, leading to the enhancement of NLRP3 and Cleaved-Caspase1 expression as well as the reduction of neurons and goblet cells. Moreover, although acacetin significantly ameliorated the depressive behavior (measured by SPT, TST, and FST) and dry eye symptoms (determined by wetted length and corneal defects) triggered by CUMS, this effect was greatly blocked by gp78 siRNA intervention (Figure 9). Taken together, these results suggest that gp78 activation is required for acacetin to prevent depression-related dry eye disease.

## Discussion

Dry eye disease represents a heterogeneous group of conditions with tear film insufficiency and symptoms of ocular surface defects, and affects tens of millions of people (Thulasi and Djalilian, 2017; Tsubota et al., 2020). Depression is an important factor involved in the development of dry eye disease (Rakofsky et al., 2021). Severity of dry eye disease is associated with symptoms of depression (Weatherby et al., 2019). Patients with depression may suffer from central sensitization which affects pain perception and pain related behavior (Wan et al., 2016). While effective management of depression can help attenuate symptoms of dry eye disease, the anti-cholinergic effect of antidepressants may contribute to dry eye symptoms due to their potential side effects on the tear film status (Wan et al., 2016; Weatherby et al., 2019). Treatment of depression with the currently used antidepressants such as selective inhibitors of serotonin receptors promotes inflammatory cytokine secretion with subsequent inflammation and apoptosis of cells on the ocular surface (Weatherby et al., 2019). Therefore, drugs targeting depression-associated dry eye disease is urgently needed (Zhang et al., 2019). Acacetin is a plant flavone with diverse therapeutic potential. Acacetin alleviates diabetes-accelerated atherosclerosis by preserving mitochondrial function *via* activating Sirtuin1 (Sirt1)/Sirtuin3 (Sirt3)/AMP-activated protein kinase (AMPK) pathway (Han et al., 2020). Acacetin also protects mice from dextran sulfate sodium-induced acute colitis through suppressing macrophage inflammation and regulating the composition of gut microbiota (Ren et al., 2020). Doxorubicin cardiomyopathy is antagonized by acacetin treatment through Sirt1-mediated activation of AMPK/nuclear factor E2-related factor 2 (Nrf2) signaling. Here, we found that acacetin rescued corneal defects and the decrease of tear production and goblet cells in CUMS mice, indicating the protective effect of acacetin in depression-associated dry eye disease.

Inflammation is an important immune response regulating the interaction between organisms and the environment, and contributes to numerous diseases, including dry eye disease (Bordoni et al., 2017). Fakih et al. (2019) observed enhanced neuro-inflammatory responses in the trigeminal ganglion, as well as increased proinflammatory markers and activated astrocytes and microglia in the trigeminal brainstem sensory complex of mice with dry eye disease. Chen et al. (2020) found NOD-like receptor 12 (NLRP12) collaborates with NLR family CARD domain-containing protein 4 (NLRC4) inflammasomes to trigger GSDMD-dependent pyroptosis and exacerbate dry eye disease. One dose of carbonized nanogels with anti-inflammatory property profoundly relieved the symptoms of dry eye disease *in vivo* (Lin et al., 2022).

The NLRP3 inflammasome is a multimeric cytosolic protein complex that controls inflammatory response and influences the process of dry eye disease (Sharma and Kanneganti, 2021). MiR-223 decreases hyperosmolarity-induced inflammation through downregulating NLRP3 activation in human corneal epithelial cells and dry eye patients (Ren et al., 2022). Calcitriol inhibits the NLRP3 inflammasome-GSDMD pyroptosis pathway leading to the alleviation of hyperosmotic stress-induced corneal epithelial cell damage in dry eye disease (Zhang et al., 2021). The upregulation of NLRP3 inflammasome is noticed in the tears and ocular surface of dry eye patients (Niu et al., 2015). The artemisinin analog β-aminoarteether maleate (SM934) mitigates dry eye disease in rodent models by regulating Toll-like Receptor 4 (TLR4)/nuclear factor (NF)-κB/NLRP3 signaling (Yang et al., 2021). Consistently, our results showed that acacetin blocked NLRP3 inflammasome/GSDMD signaling pathway and reduced the concentrations of proinflammatory cytokines TNF-α, IL-1β, and IL-18, supporting the essential role of NLRP3 inflammasome-mediated inflammation in the development and treatment of dry eye disease.

Ubiquitination is a type of posttranslational modification of intracellular proteins, in which the ubiquitin moiety is covalently attached to a target protein to influence protein stability, interaction partner and biological function (Gu and Jan 2020). Glycoprotein 78 (gp78) is an E3 ubiquitin ligase within the endoplasmic reticulum-associated degradation pathway, which plays an essential role in multiple physiological and pathological processes (Joshi et al., 2017). Deacetylation of heat shock protein A5 (HSPA5) by histone deacetylase6 (HDAC6) results in gp78-mediated HSPA5 ubiquitination at K447 and suppresses metastasis of breast cancer (Chang et al., 2016). High expression levels of gp78 are related to poor outcomes in both endoplasmic reticulum (ER)-positive and ER-negative tumors (Singhal et al., 2022). Cyclin-dependent kinase5 (CDK5)-mediated phosphorylation-dependent ubiquitination and degradation of E3 ubiquitin ligases gp78 accelerates neuronal death in Parkinson’s disease (Wang et al., 2018). Xu et al. (2022) reported that gp78 mediates the ubiquitination of NLRP3, which suppresses NLRP3 inflammasome activation by inhibiting the oligomerization and subcellular translocation of NLRP3. In our study, the upregulation of gp78/Insig-1 elicited by acacetin promoted NLRP3 ubiquitination and attenuated CUMS-induced inflammatory response and dry eye disease in mice; however, interference of gp78 blunted acacetin-conferred beneficial effects in CUMS-related dry eye disease, confirming the crucial role of gp78 in regulating NLRP3 ubiquitination-regulated inflammation and dry eye disease in depressive rodents.

To conclude, the present study demonstrated that acacetin prevents depression-associated dry eye disease through suppression of NLRP3 ubiquitination-mediated inflammatory response *via* gp78 signaling. Our results suggest that acacetin may be a powerful therapeutic candidate for depression-related dry eye disease.

## Data Availability

The original contributions presented in the study are included in the article/Supplementary Material, further inquiries can be directed to the corresponding authors.
