# Supplementary figures and images for: Acacetin protects against depression-associated dry eye disease by regulating ubiquitination of NLRP3 through gp78 signal
Source: Front Pharmacol. 2022 Oct 10;13:984475. doi: 10.3389/fphar.2022.984475 (PMC9588975; doi:10.3389/fphar.2022.984475)

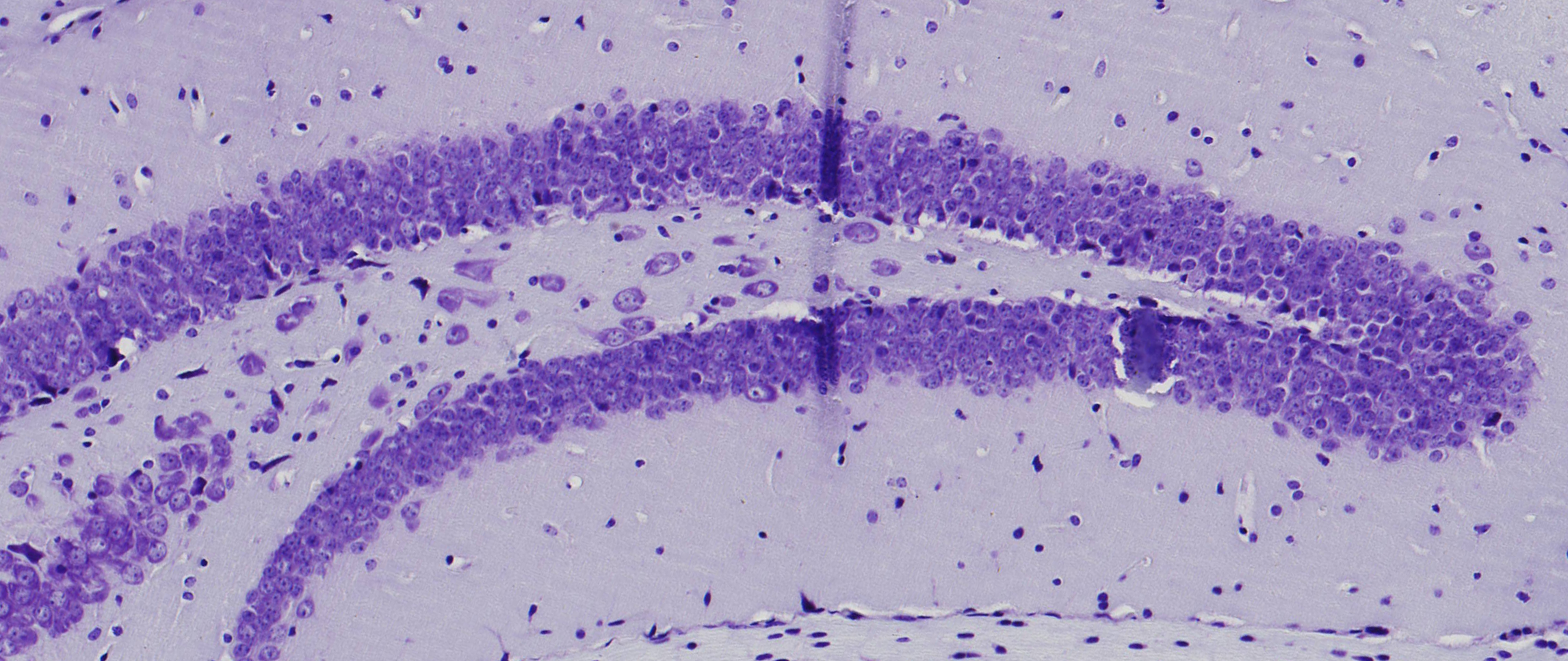

Supplement: Supplementary file 1 [file DataSheet3.ZIP › 2022-0705-DES depression -original data 3/Nissl/CUMS.tif]

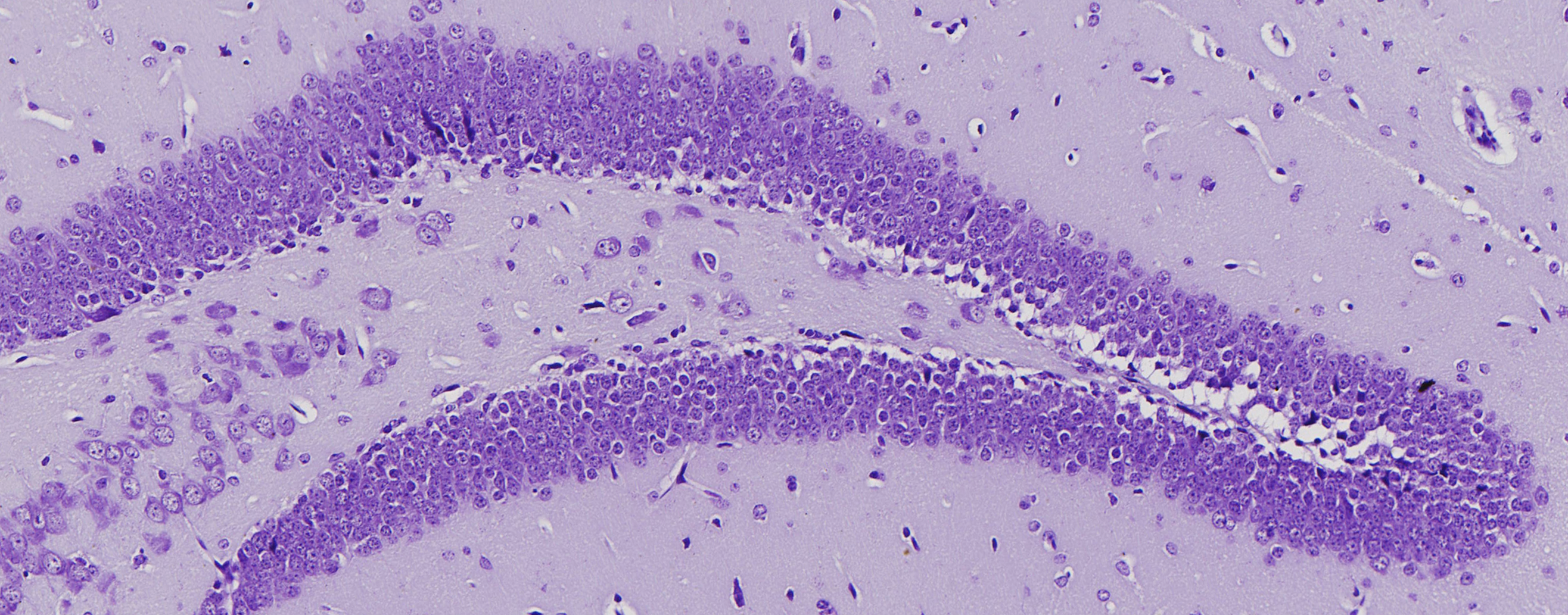

Supplement: Supplementary file 1 [file DataSheet3.ZIP › 2022-0705-DES depression -original data 3/Nissl/CUMS+Acacetin-H.tif]

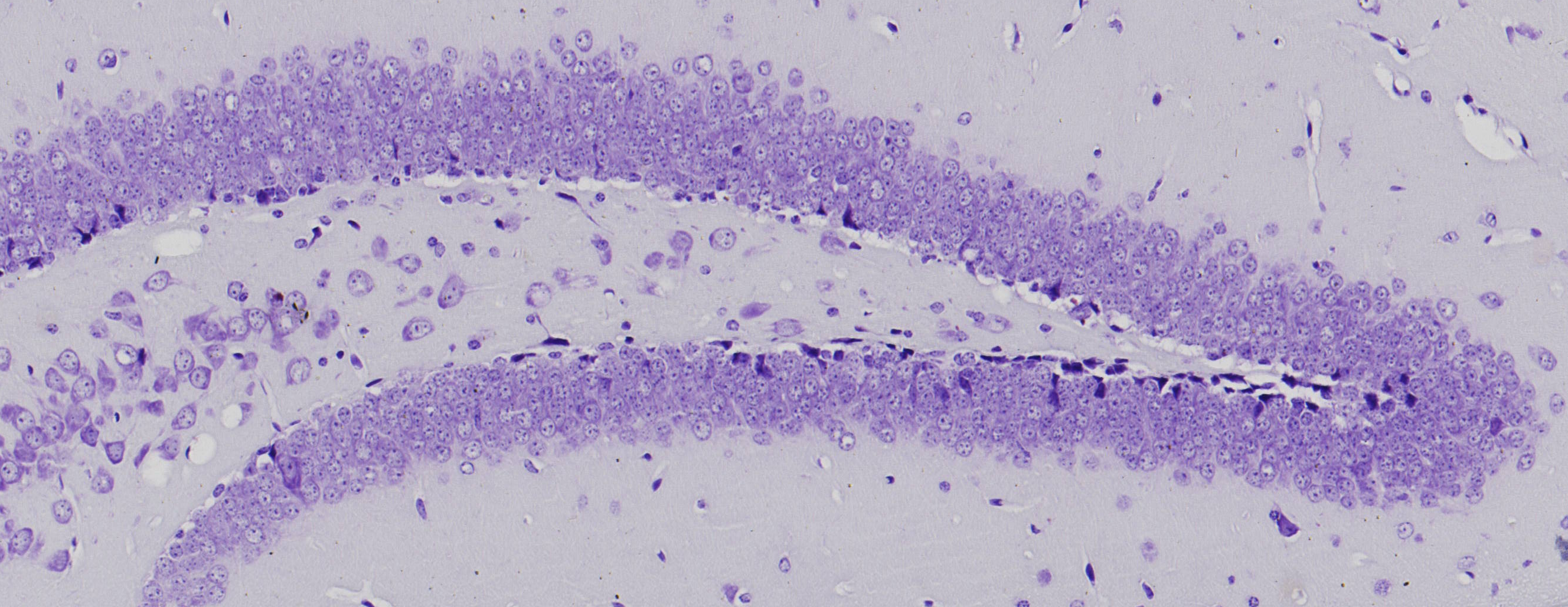

Supplement: Supplementary file 1 [file DataSheet3.ZIP › 2022-0705-DES depression -original data 3/Nissl/CUMS+si-gp78.tif]

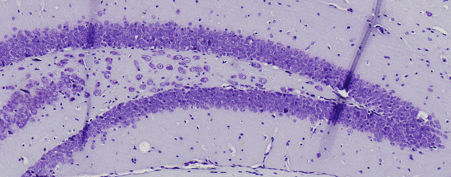

Supplement: Supplementary file 1 [file DataSheet3.ZIP › 2022-0705-DES depression -original data 3/Nissl/CUMS+si-gp78+Acacetin-H.tif]

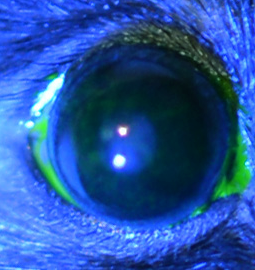

Supplement: Supplementary file 3 [file DataSheet2.ZIP › 2022-0705-DES depression -original data 2/fluorescent(FL)/Acacetin-H.tif]

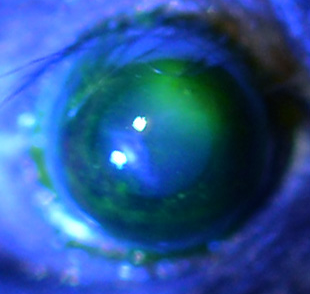

Supplement: Supplementary file 3 [file DataSheet2.ZIP › 2022-0705-DES depression -original data 2/fluorescent(FL)/Acacetin-L.tif]

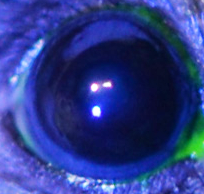

Supplement: Supplementary file 3 [file DataSheet2.ZIP › 2022-0705-DES depression -original data 2/fluorescent(FL)/Control.tif]

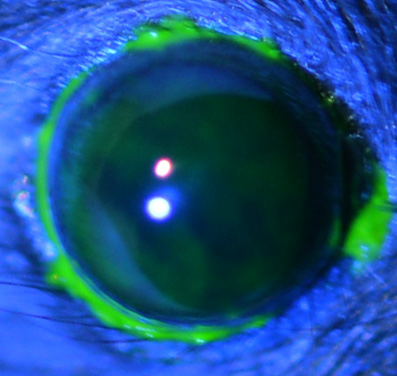

Supplement: Supplementary file 3 [file DataSheet2.ZIP › 2022-0705-DES depression -original data 2/fluorescent(FL)/CUMS.tif]

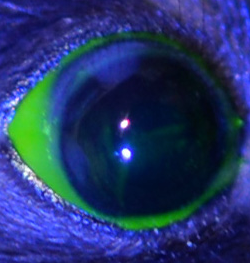

Supplement: Supplementary file 3 [file DataSheet2.ZIP › 2022-0705-DES depression -original data 2/fluorescent(FL)/CUMS+Acacetin-H.tif]

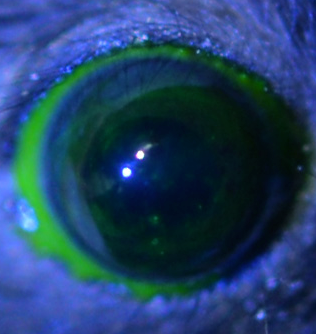

Supplement: Supplementary file 3 [file DataSheet2.ZIP › 2022-0705-DES depression -original data 2/fluorescent(FL)/CUMS+si-gp78.tif]

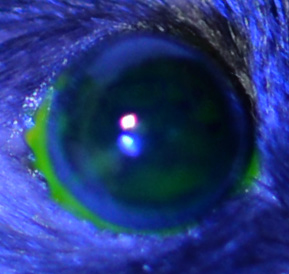

Supplement: Supplementary file 3 [file DataSheet2.ZIP › 2022-0705-DES depression -original data 2/fluorescent(FL)/CUMS+si-gp78+Acacetin-H.tif]

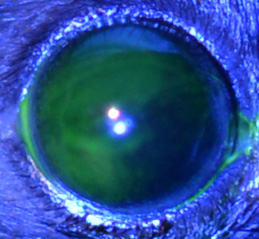

Supplement: Supplementary file 3 [file DataSheet2.ZIP › 2022-0705-DES depression -original data 2/fluorescent(FL)/Escitalopram.tif]

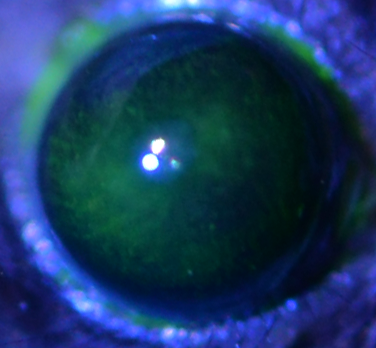

Supplement: Supplementary file 3 [file DataSheet2.ZIP › 2022-0705-DES depression -original data 2/fluorescent(FL)/Vehicle.tif]

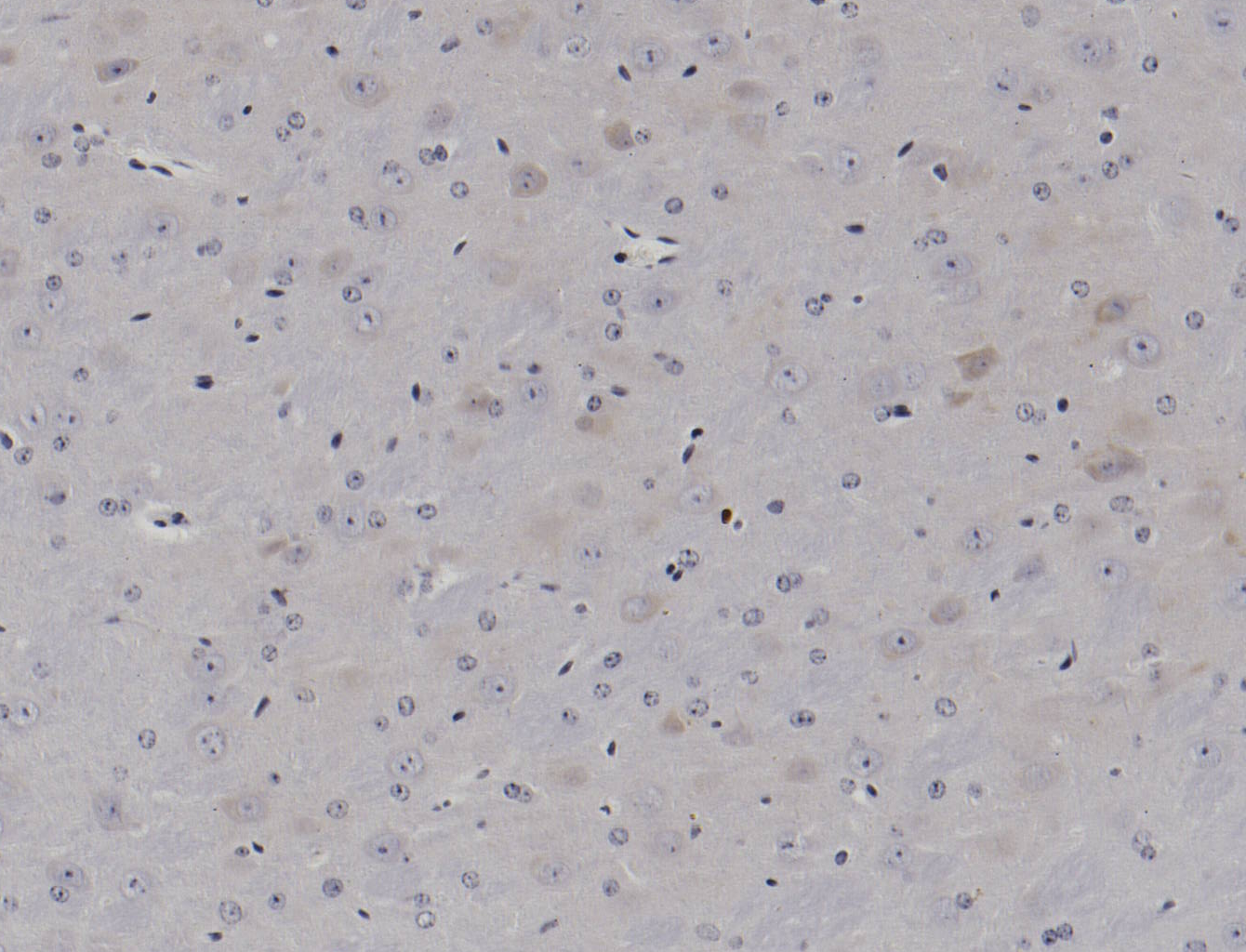

Supplement: Supplementary file 3 [file DataSheet2.ZIP › 2022-0705-DES depression -original data 2/NLRP3/CUMS.tif]

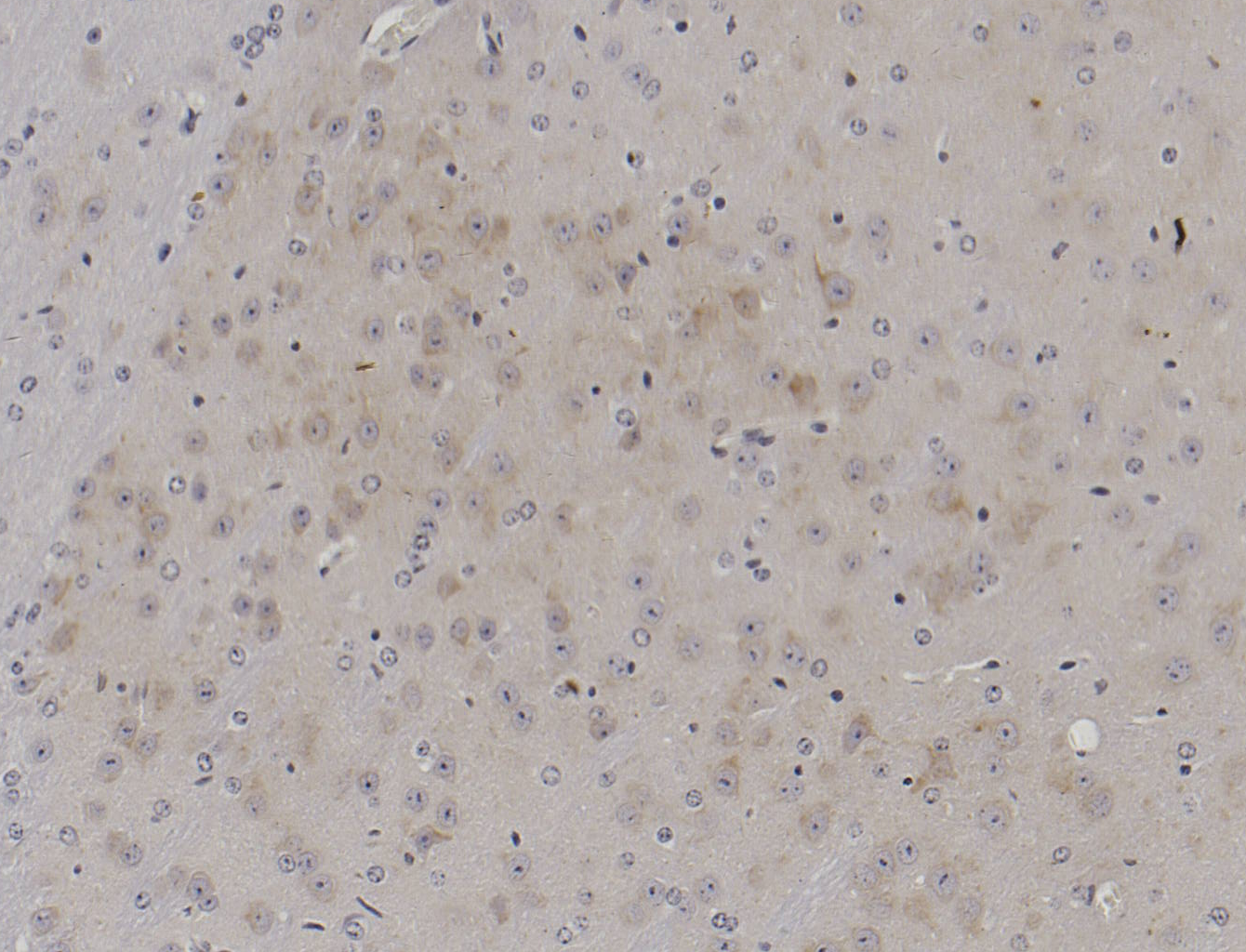

Supplement: Supplementary file 3 [file DataSheet2.ZIP › 2022-0705-DES depression -original data 2/NLRP3/CUMS+Acacetin-H.tif]

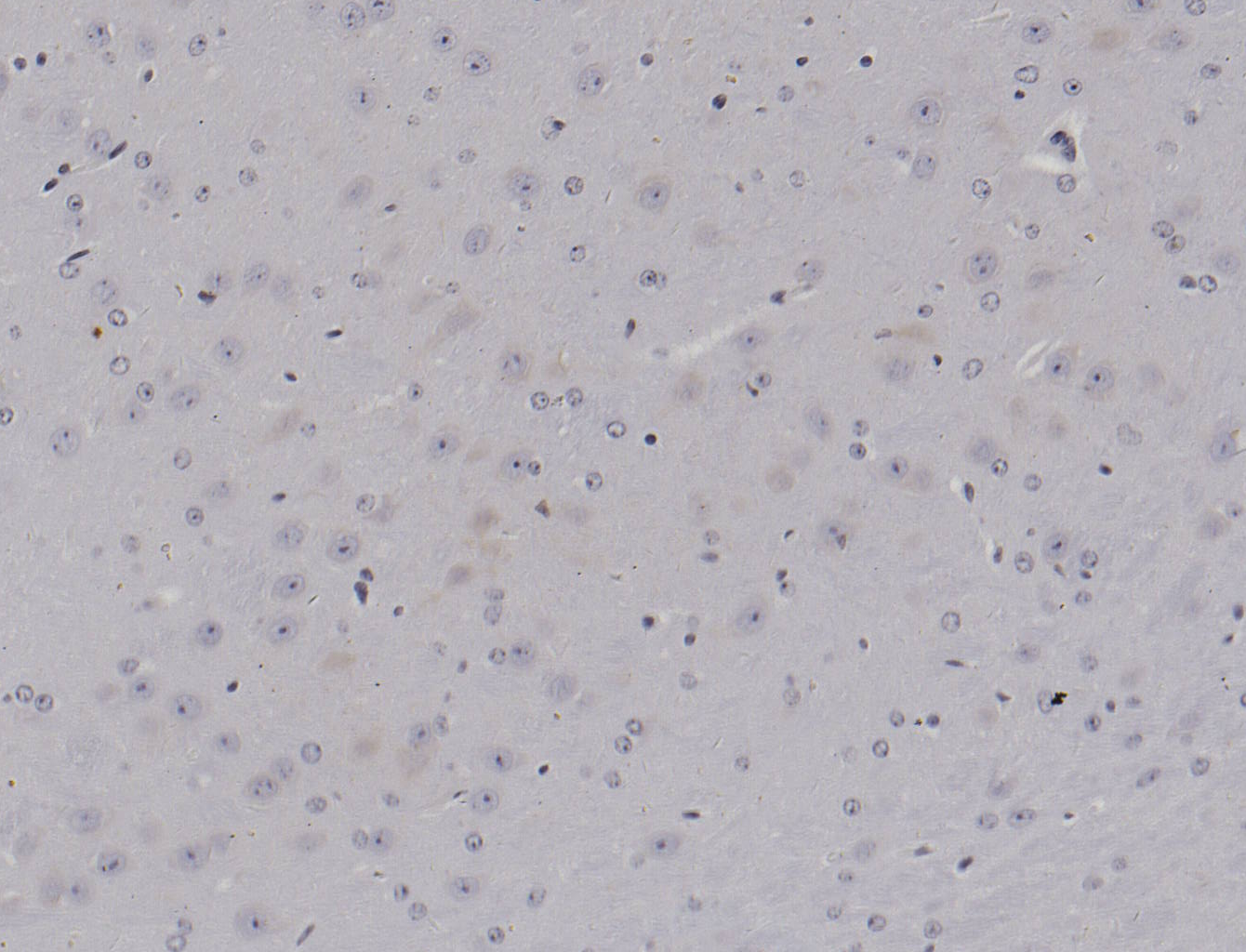

Supplement: Supplementary file 3 [file DataSheet2.ZIP › 2022-0705-DES depression -original data 2/NLRP3/CUMS+si-gp78.tif]

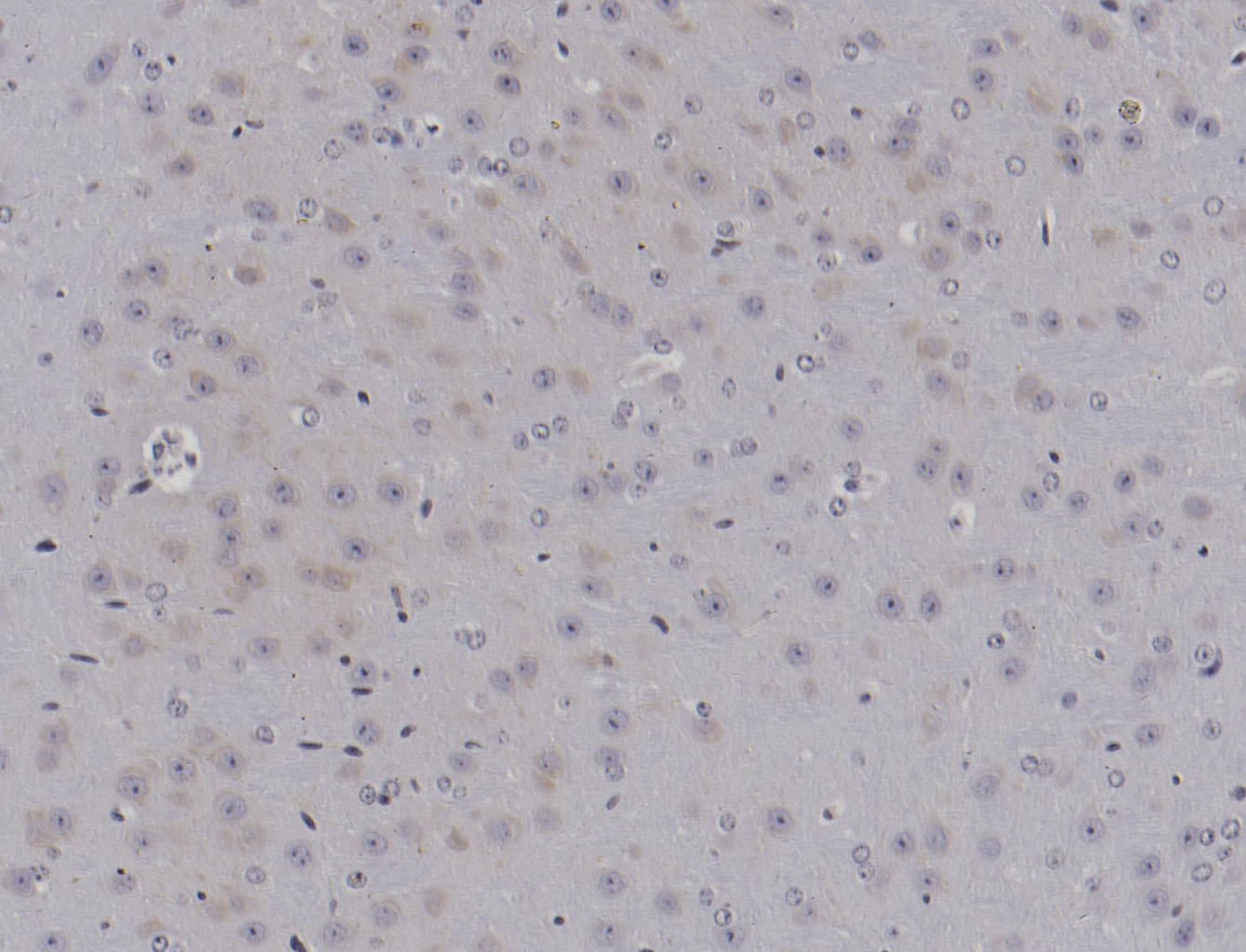

Supplement: Supplementary file 3 [file DataSheet2.ZIP › 2022-0705-DES depression -original data 2/NLRP3/CUMS+si-gp78+Acacetin-H.tif]

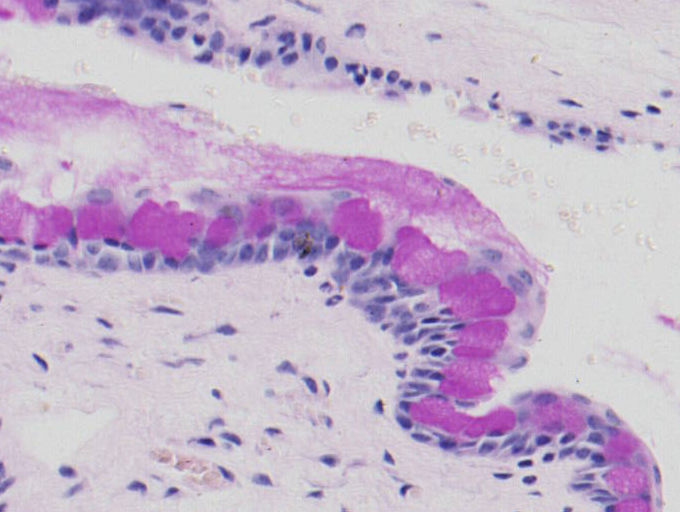

Supplement: Supplementary file 3 [file DataSheet2.ZIP › 2022-0705-DES depression -original data 2/PAS/Acacetin-H.tif]

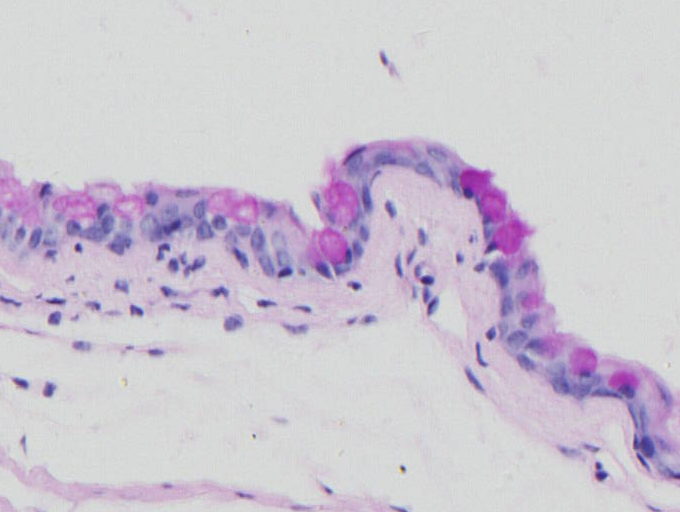

Supplement: Supplementary file 3 [file DataSheet2.ZIP › 2022-0705-DES depression -original data 2/PAS/Acacetin-L.tif]

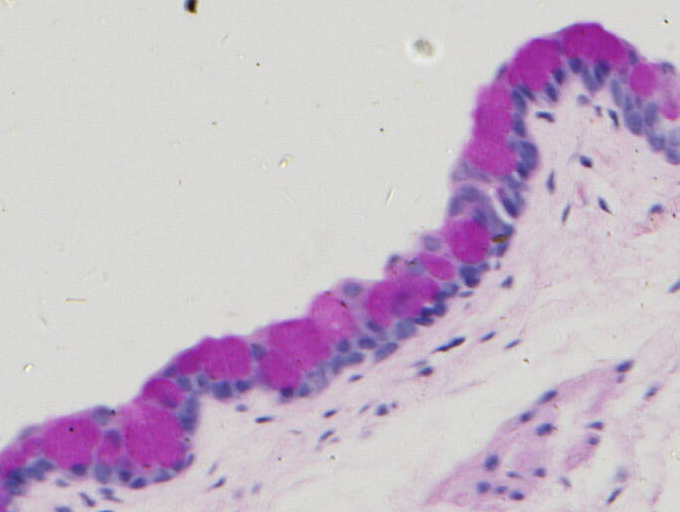

Supplement: Supplementary file 3 [file DataSheet2.ZIP › 2022-0705-DES depression -original data 2/PAS/Control.tif]

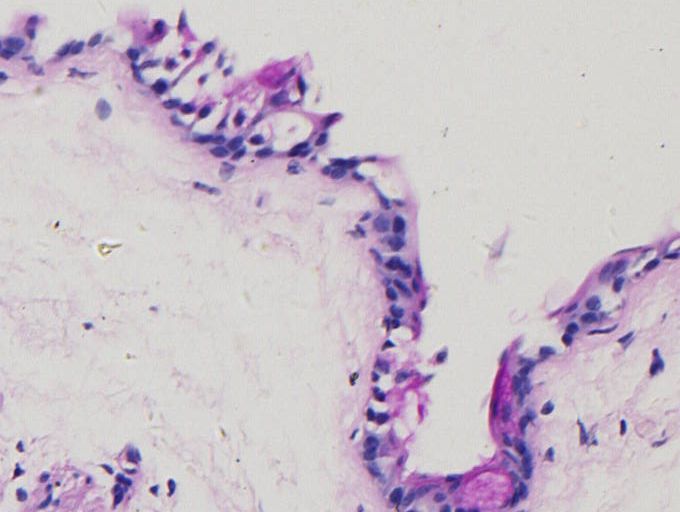

Supplement: Supplementary file 3 [file DataSheet2.ZIP › 2022-0705-DES depression -original data 2/PAS/CUMS.tif]

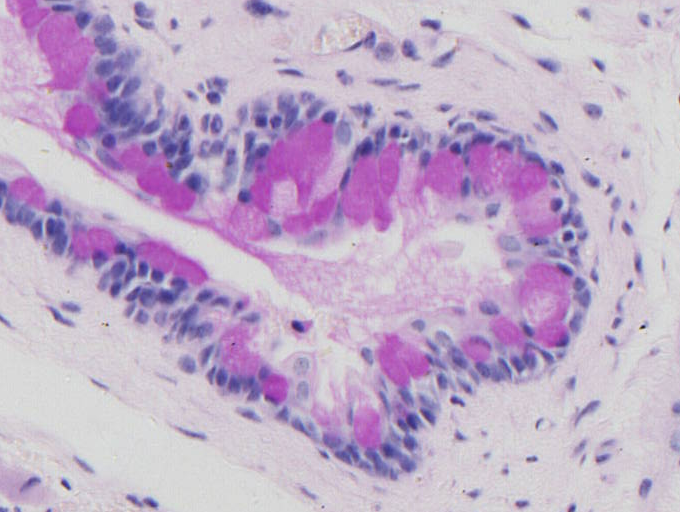

Supplement: Supplementary file 3 [file DataSheet2.ZIP › 2022-0705-DES depression -original data 2/PAS/CUMS+Acacetin-H.tif]

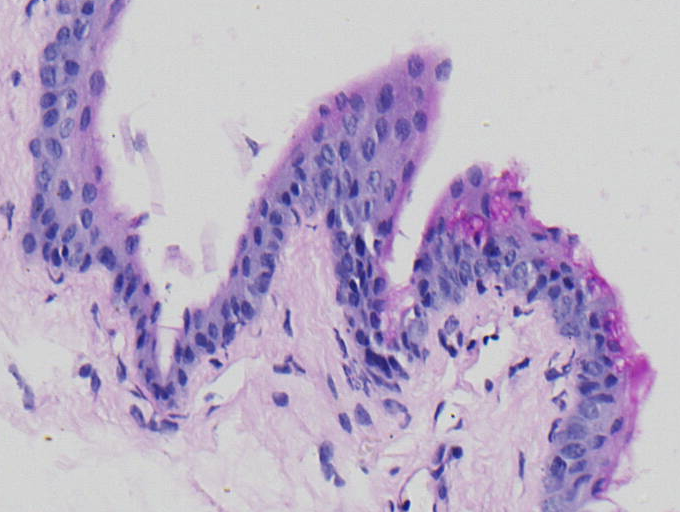

Supplement: Supplementary file 3 [file DataSheet2.ZIP › 2022-0705-DES depression -original data 2/PAS/CUMS+si-gp78.tif]

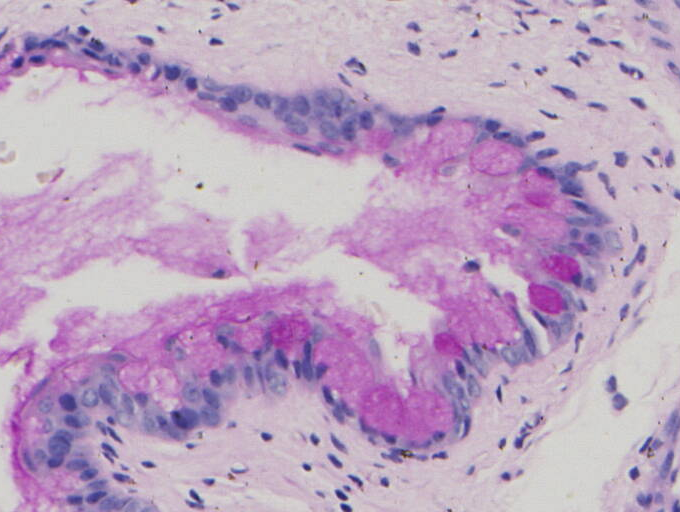

Supplement: Supplementary file 3 [file DataSheet2.ZIP › 2022-0705-DES depression -original data 2/PAS/CUMS+si-gp78+Acacetin-H.tif]

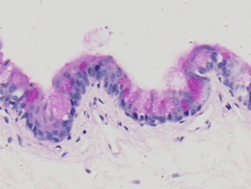

Supplement: Supplementary file 3 [file DataSheet2.ZIP › 2022-0705-DES depression -original data 2/PAS/Escitalopram.tif]

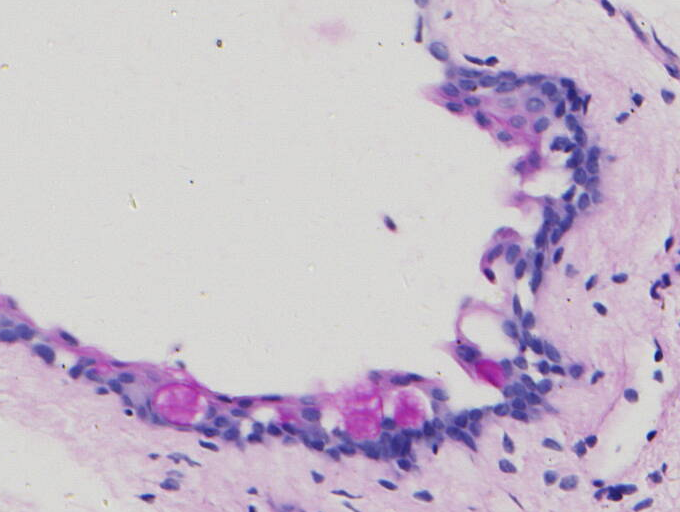

Supplement: Supplementary file 3 [file DataSheet2.ZIP › 2022-0705-DES depression -original data 2/PAS/Vehicle.tif]
