# Supplementary material for: Acacetin protects against depression-associated dry eye disease by regulating ubiquitination of NLRP3 through gp78 signal
Source: Front Pharmacol. 2022 Oct 10;13:984475. doi: 10.3389/fphar.2022.984475 (PMC9588975; doi:10.3389/fphar.2022.984475)
Supplement: Supplementary file 2 [file DataSheet1.ZIP › 2022-0705-DES depression-original data 1/2022-0629-Western Blot.pptx]

## Slide 1
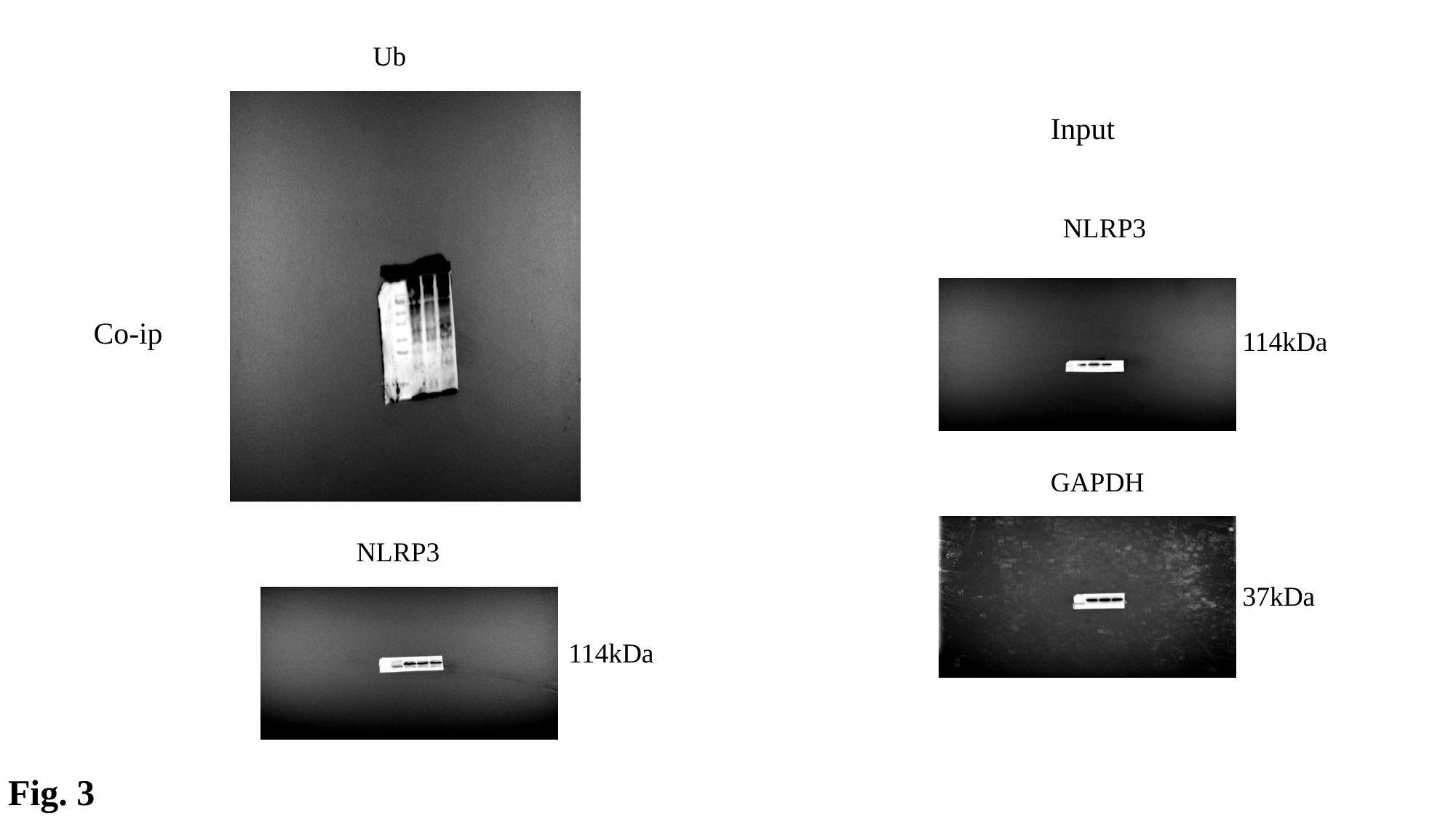

Ub
Input
NLRP3
Co-ip
114kDa
GAPDH
NLRP3
37kDa
114kDa
Fig. 3

## Slide 2
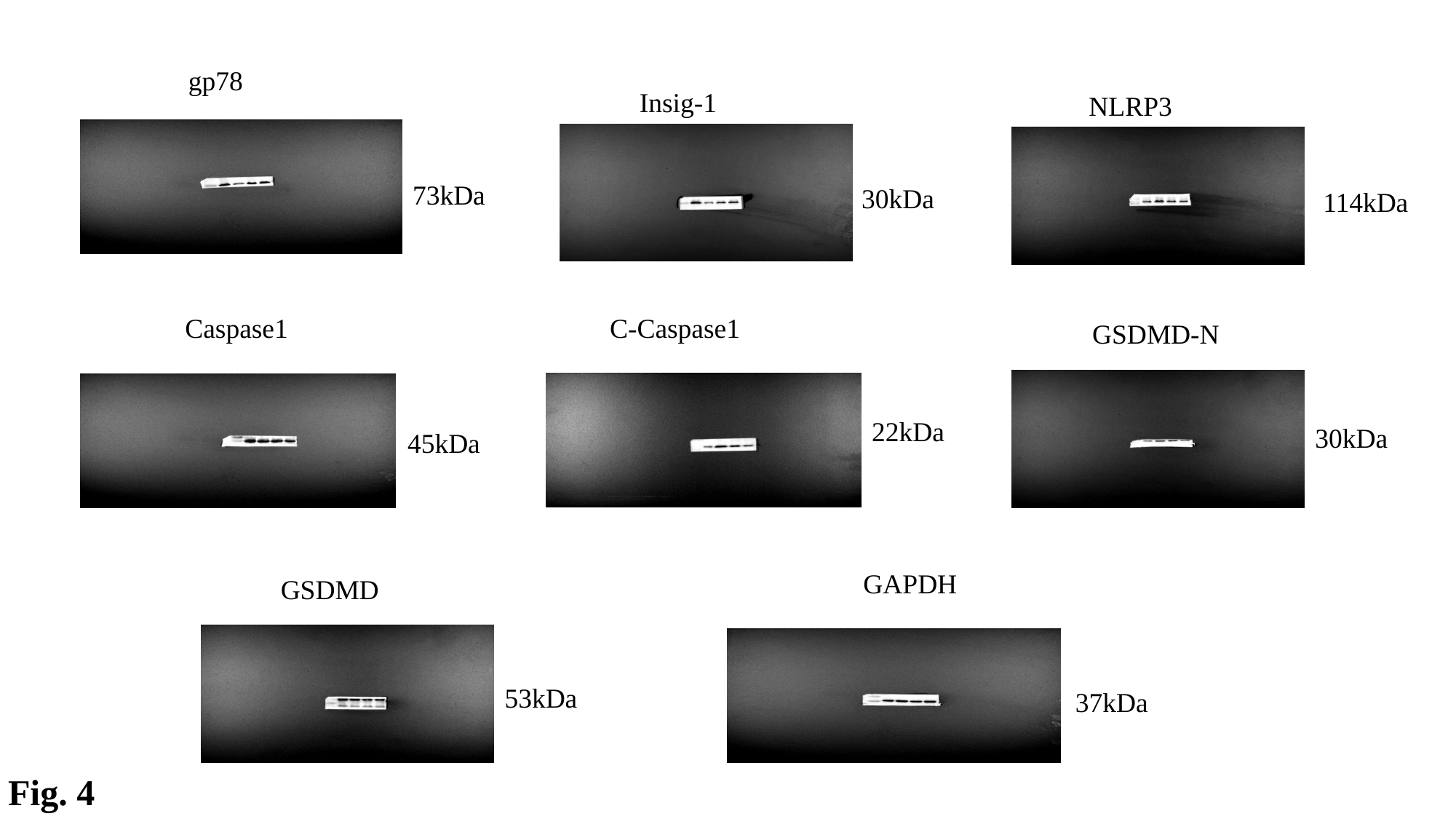

gp78
73kDa
Insig-1
30kDa
NLRP3
114kDa
C-Caspase1
22kDa
Caspase1
45kDa
GSDMD-N
30kDa
GAPDH
37kDa
GSDMD
53kDa
Fig. 4

## Slide 3
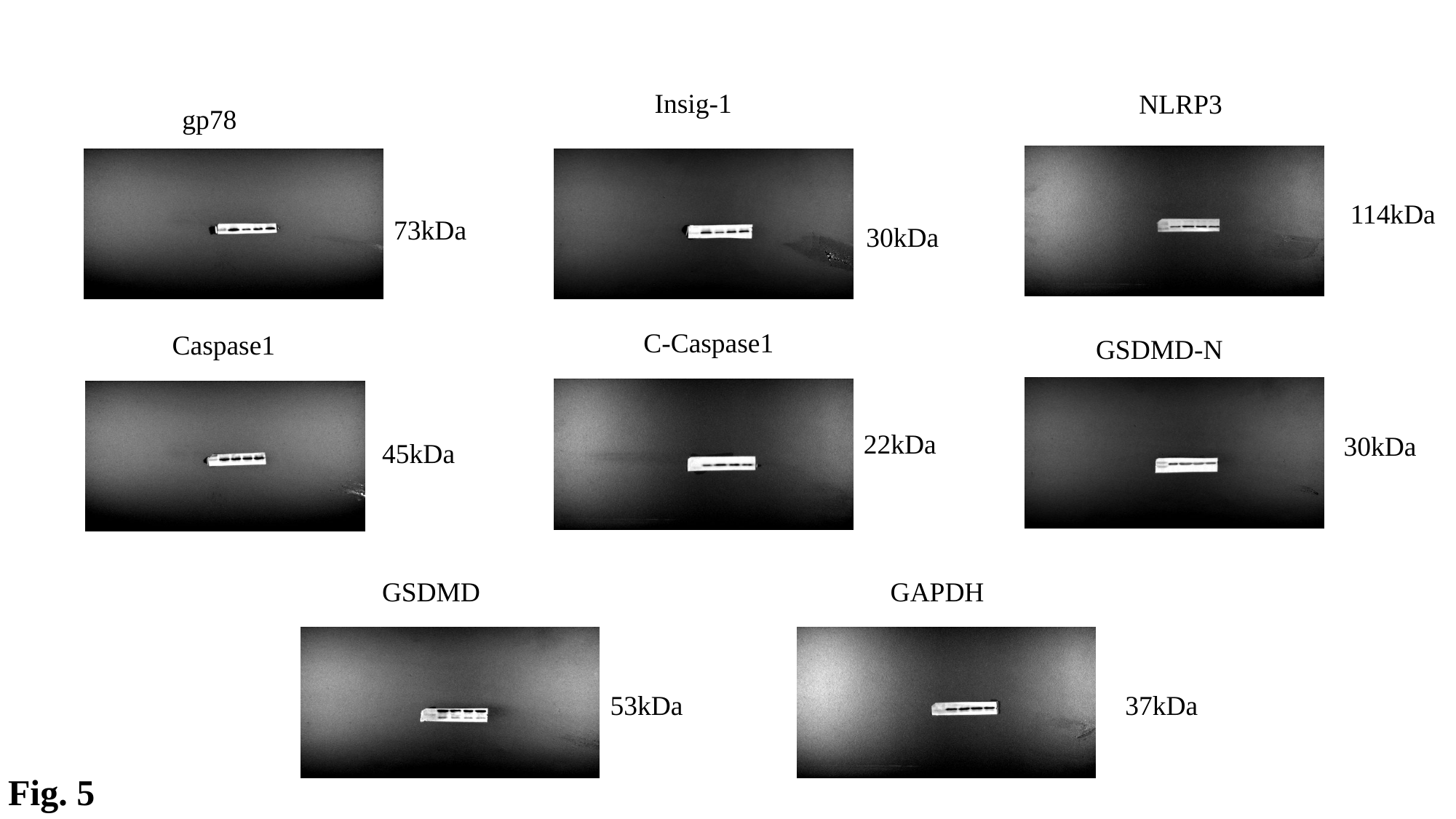

Insig-1
NLRP3
gp78
114kDa
73kDa
30kDa
C-Caspase1
Caspase1
GSDMD-N
22kDa
30kDa
45kDa
GSDMD
GAPDH
53kDa
37kDa
Fig. 5

## Slide 4
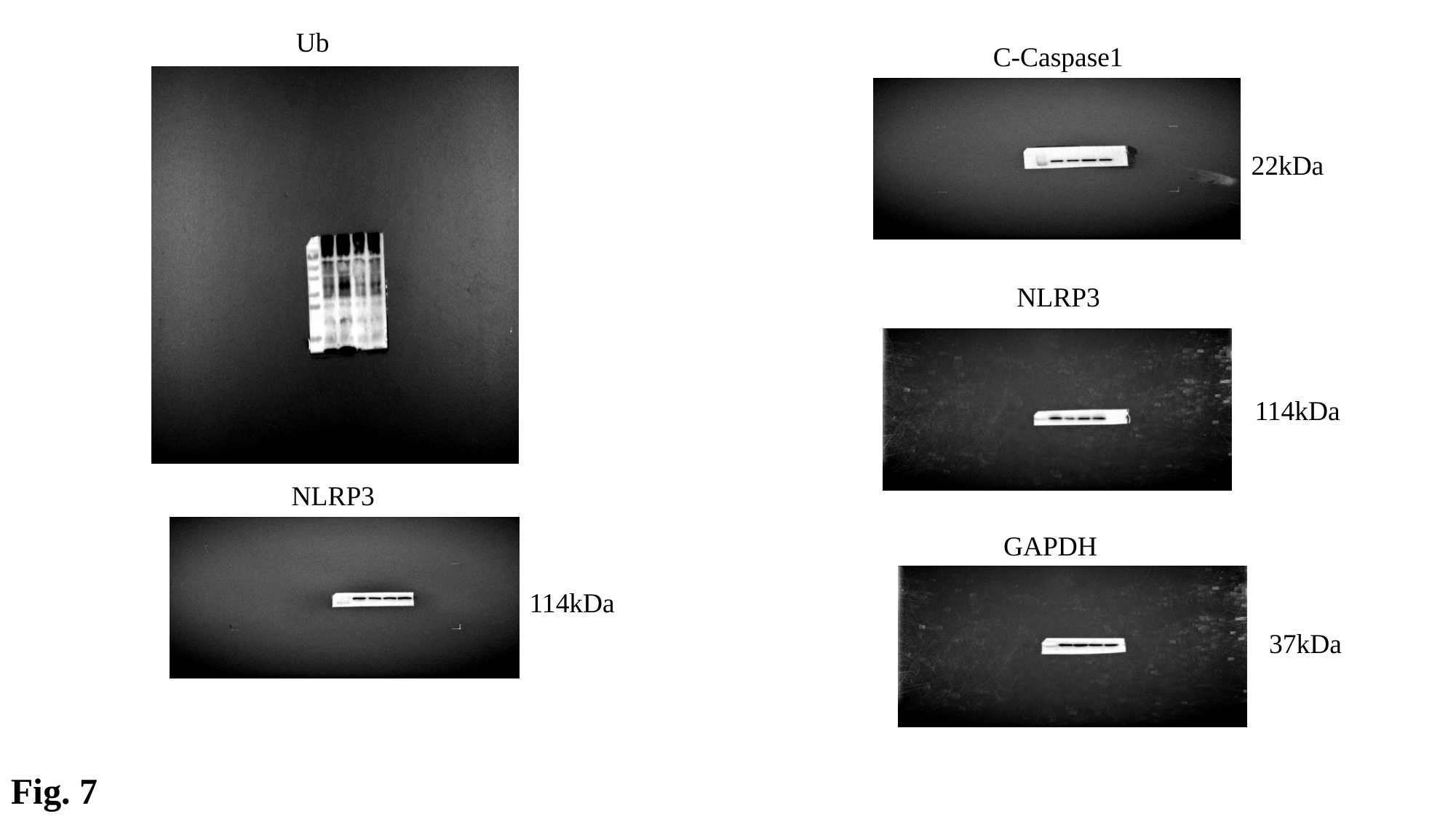

Ub
C-Caspase1
22kDa
NLRP3
114kDa
NLRP3
GAPDH
37kDa
114kDa
Fig. 7

## Slide 5
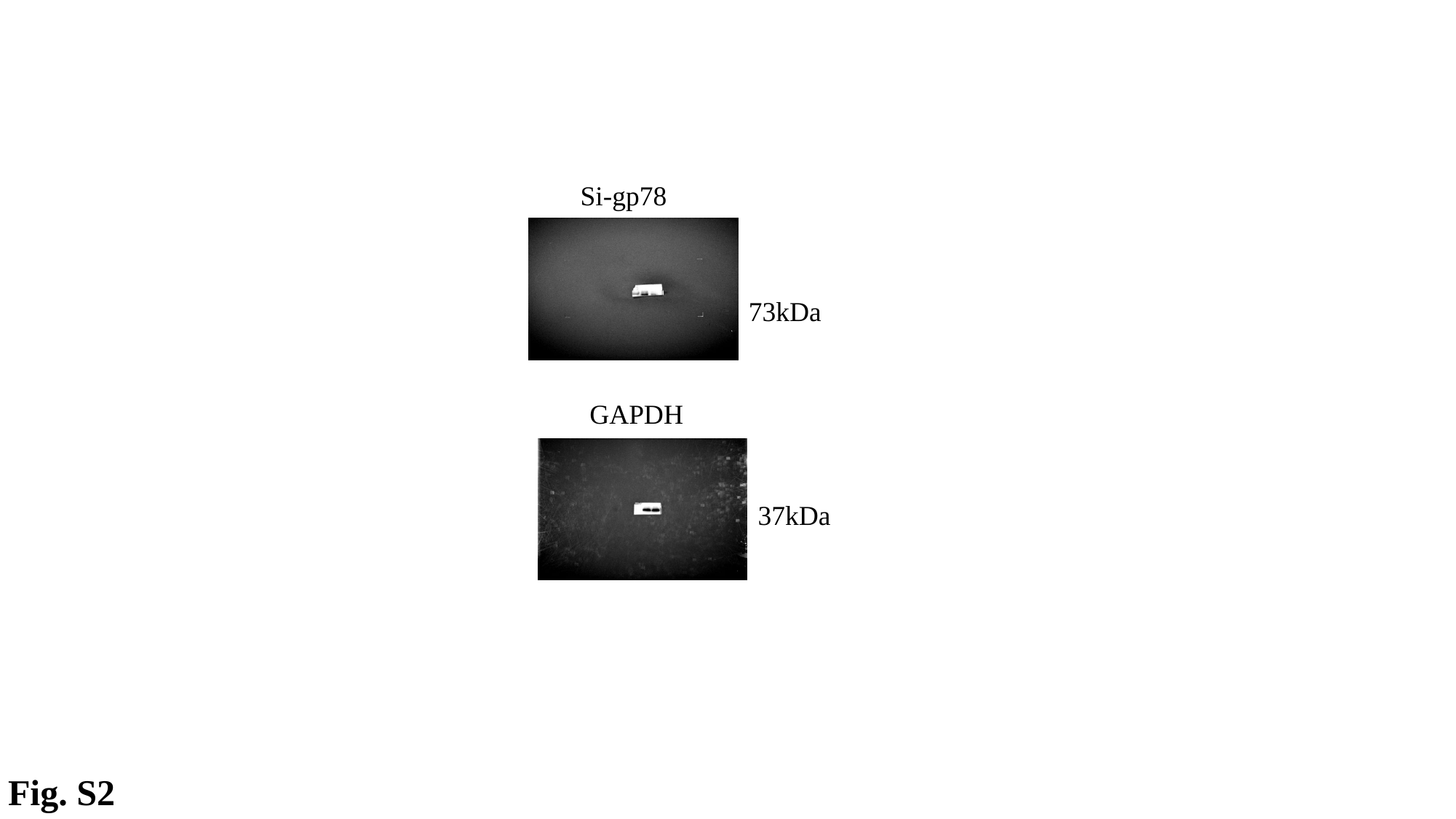

Si-gp78
73kDa
GAPDH
37kDa
Fig. S2
