# Supplementary material for: Acacetin protects against depression-associated dry eye disease by regulating ubiquitination of NLRP3 through gp78 signal
Source: Front Pharmacol. 2022 Oct 10;13:984475. doi: 10.3389/fphar.2022.984475 (PMC9588975; doi:10.3389/fphar.2022.984475)
Supplement: Supplementary file 4 [file Image1.pdf]

Supplementary figures

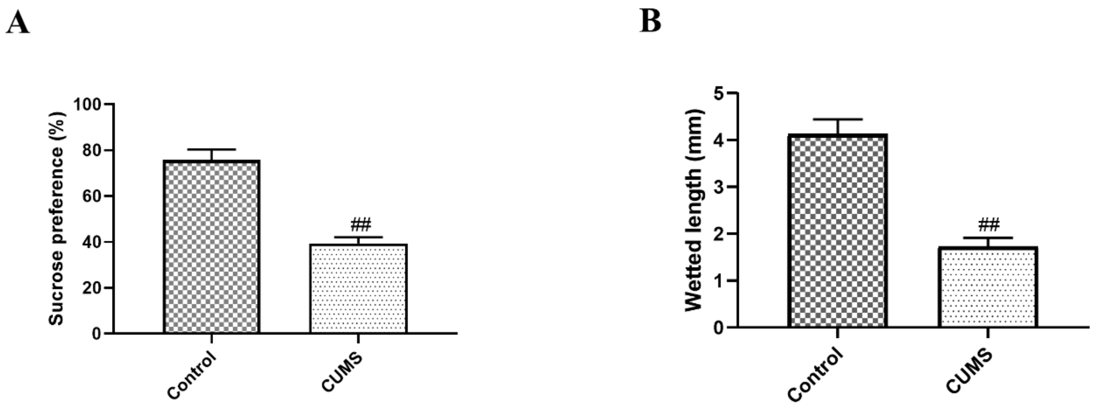

Fig. S1. Sucrose preference ratio and tear production after 4 weeks of chronic unpredictable mild stress (CUMS) modeling.

(A) Sucrose preference ratio of the experimental groups following 4 weeks of CUMS modeling. (B) Tear production of the experimental groups after 4 weeks of CUMS modeling. Results are expressed as the mean ± SEM (Control, n = 9; CUMS, n = 36 per group).

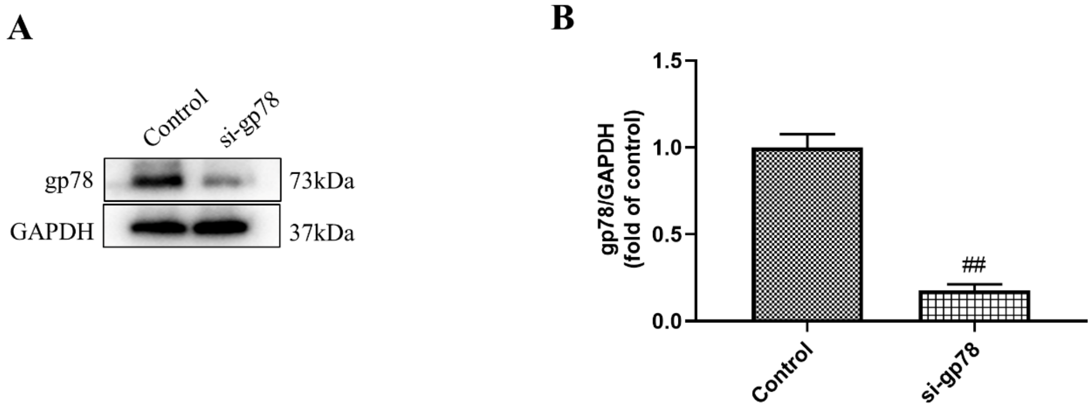

Fig. S2. Characterization of gp78 siRNA vectors by western blotting.
(A) Representative western blot of gp78 siRNA. (B) Quantitative analysis of gp78 siRNA protein expression. Results are expressed as the mean ± SEM (n = 3 per group).
